# Racemases and epimerases operating through a 1,1-proton transfer mechanism: reactivity, mechanism and inhibition

**DOI:** 10.1039/d0cs00540a

**Published:** 2021-04-12

**Authors:** Matthew D. Lloyd, Maksims Yevglevskis, Amit Nathubhai, Tony D. James, Michael D. Threadgill, Timothy J. Woodman

**Affiliations:** Drug & Target Discovery, Department of Pharmacy & Pharmacology, University of Bath Claverton Down Bath BA2 7AY UK M.D.Lloyd@bath.ac.uk +44-(0)1225-386786; CatSci Ltd., CBTC2 Capital Business Park Wentloog Cardiff CF3 2PX UK; University of Sunderland, School of Pharmacy & Pharmaceutical Sciences, Sciences Complex Sunderland SR1 3SD UK; Department of Chemistry, University of Bath Claverton Down Bath BA2 7AY UK; School of Chemistry and Chemical Engineering, Henan Normal University Xinxiang 453007 People's Republic of China; Institute of Biological, Environmental & Rural Sciences, Aberystwyth University Aberystwyth SY23 3BY UK

## Abstract

Racemases and epimerases catalyse changes in the stereochemical configurations of chiral centres and are of interest as model enzymes and as biotechnological tools. They also occupy pivotal positions within metabolic pathways and, hence, many of them are important drug targets. This review summarises the catalytic mechanisms of PLP-dependent, enolase family and cofactor-independent racemases and epimerases operating by a deprotonation/reprotonation (1,1-proton transfer) mechanism and methods for measuring their catalytic activity. Strategies for inhibiting these enzymes are reviewed, as are specific examples of inhibitors. Rational design of inhibitors based on substrates has been extensively explored but there is considerable scope for development of transition-state mimics and covalent inhibitors and for the identification of inhibitors by high-throughput, fragment and virtual screening approaches. The increasing availability of enzyme structures obtained using X-ray crystallography will facilitate development of inhibitors by rational design and fragment screening, whilst protein models will facilitate development of transition-state mimics.

## Abstract 2

Racemases and epimerases using a deprotonation/reprotonation mechanism are important drug targets and have important biotechnological applications. This review focuses on the reactivity, mechanism, and inhibition of these versatile enzymes.

## Graphical abstract

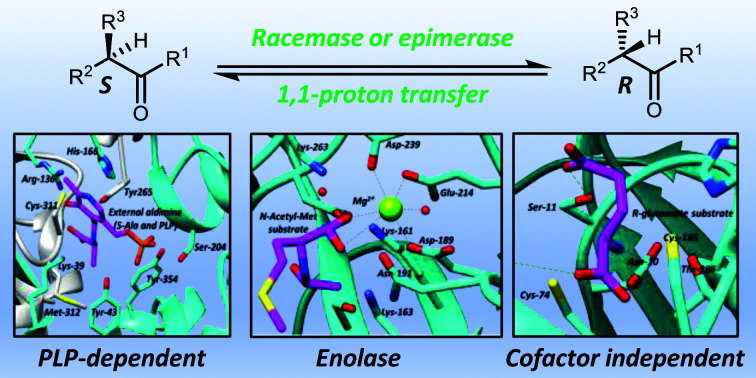

## Introduction

Chirality is at the very heart of Chemical Biology. Proteins, nucleic acids, carbohydrates and many lipids are all chiral molecules, as are the overwhelming majority of their monomer precursors. In addition, many cellular metabolites also contain chiral centres. It is well-known that, for most chiral biomolecules, one particular configuration is preferred; thus proteins contain predominantly chiral amino-acids with l-configuration^1,2^ (*S*-configuration in the Cahn-Ingold-Prelog system^3^ except for *R*-cysteine and achiral glycine). Similarly, carbohydrates are or contain predominantly d-sugars, with l-ascorbic acid (vitamin C) being a well-known exception. An important consequence of the chiral nature of proteins is that, when they interact with other chiral molecules, a diastereomeric situation arises; thus, most proteins will be highly selective for a particular configuration of their interacting partners (substrate, inhibitor, allosteric effector). An important consequence of this is that different stereoisomers of chiral drugs are effectively different drugs, which will generally have different protein targets (enzyme, receptors *etc.*) and different pharmacokinetics.^4,5^ Finally, many drugs are known to undergo metabolic changes of chiral configuration *in vivo*,^4,5^*e.g.* ibuprofen and related ‘profens’ (reviewed in ref. 6–8) and mandelic acid.^9,10^ In addition the 2-(aryloxy)propanoic acid herbicides undergo changes in chiral configuration which are mediated by soil bacteria.^11–13^

Notwithstanding the fact that most biological molecules exist overwhelmingly in one stereochemical configuration, there are many examples where minor stereoisomers play an essential role. The most well-known example of this is proteinogenic amino-acids such as alanine and glutamate, which are found in their d-configuration (*R*-configuration) within bacterial peptidoglycan.^14–17^ In most cases, these minor stereoisomers are not biosynthesised *de novo* but are obtained by changing the stereochemical configuration of the most abundant isomer into that of the less abundant isomer.

The enzymes which perform these changes in stereochemical configuration are known as racemases and epimerases, which have been shown to have a pivotal position in metabolism, and thus have gained significant interest as drug targets for diseases such as bacterial infections,^14,18–25^ Chagas disease,^26–28^ cancer,^6,7,18,29^ Alzheimer's disease and other dementias,^1,2,30–32^ formation of cataracts^1,33^ and diabetic retinopathy;^34^ racemase levels are also a marker of ischaemic stroke.^35^ Inhibition of diaminopimelate epimerase activity also potentiates cephem antibiotic activity by compromising the integrity of the bacterial cell wall.^36^

Low activity or concentrations of racemases/epimerases (AMACR,^37^ methylmalonyl-CoA epimerase^38^) are associated with inherited errors in metabolism and may also be associated with stroke and dementia^39^ and neurodegenerative diseases,^40^ such as Amyotrophic Lateral Sclerosis (ALS, a.k.a. motor neurone disease, Lou Gehrig's disease). Increased methylmalonic acid levels in the aging population (resulting from a decrease in methylmalonyl-CoA epimerase activity) is suggested to promote an aggressive cancer phenotype by upregulation of the SOX4 transcription factor.^41^ Increased levels of aspartate/glutamate racemases protect *Salmonella enterica* from aminoacrylate metabolic stress.^42^ Increased activity of the bifunctional enzyme UDP-*N*-acetylglucosamine 2-epimerase/*N*-acetylmannosamine kinase results in sialuria, an extremely rare genetic disorder, while knockout of the corresponding gene is lethal in mice.^43,44^ Mutations in this epimerase are linked to hereditary inclusion body myopathy (HIBM).^44,45^ In addition, *O*-ureidoserine racemase is involved in the biosynthesis of the antibiotic d-cycloserine,^46^ while a peptide epimerase is found in funnel web spider (*Agelenopsis aperta*) venom which interconverts two 48 amino-acid peptides differing only in the configuration at a single serine residue (Ser-46).^47^ Finally, racemases and epimerases are used in dynamic kinetic resolutions and other biotechnological applications.^48–54^

Racemases and epimerases use several different strategies to bring about changes in stereochemical configuration of their substrates, including the use of radical reactions,^55–60^ elimination and re-addition of nucleotides^20,61^ and the use of redox cofactors.^18,19,62–64^ An important example of a ‘epimerase’ utilising redox cofactors is decaprenylphosphoryl-β-d-ribose epimerase (DprE); however, this is not a true epimerase reaction as the oxidative and reductive reactions are catalysed by separate enzymes (DprE1 and 2, respectively) using different cofactors [flavin adenine dinucleotide (oxidised) and nicotinamide adenine dinucleotide (reduced)].^65–68^

**Scheme 1:**
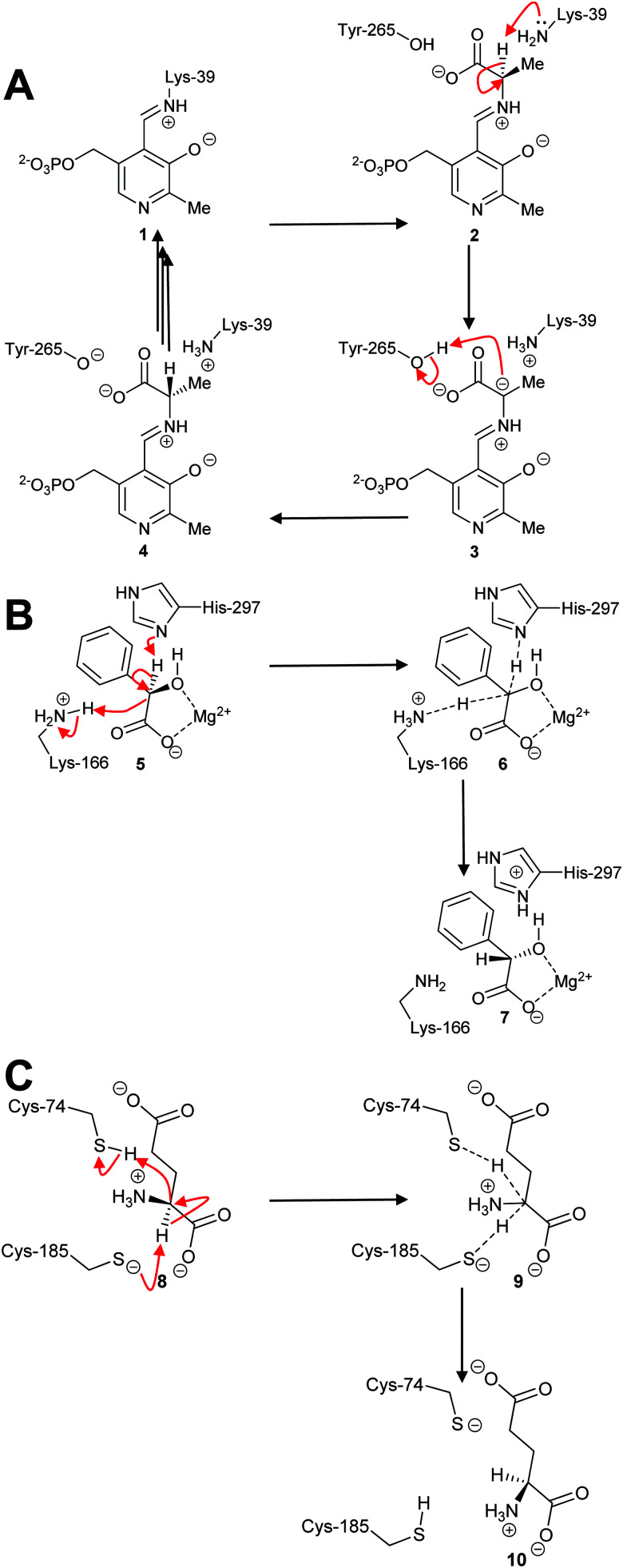
Example mechanisms of racemases and epimerases operating by a 1,1-proton transfer mechanism. (A) PLP-dependent amino acid racemases, as shown by alanine racemase;^5,69^ (B) Metal-dependent (enolase) enzymes, as shown by mandelate racemase;^74,75^ (C) Cofactor-independent racemases as shown by glutamate racemase.^63,82,83^ Dashed lines show bonds being broken or formed in the transition state.

By far the most common mechanism used by racemases and epimerases is the deprotonation/reprotonation^14,18,20,63^ (1,1-proton transfer) reaction. These enzymes fall into three classes: those which are pyridoxal 5′-phosphate (PLP)-dependent;^5,50,69,70^ those which use metal ions (enolase enzymes^14,49,71,72^); and those which are cofactor-independent (Scheme 1).^6,7,18,20,63^ The PLP-dependent enzymes (Scheme 1A) catalyse exchange between PLP in the internal aldimine **1** (catalytic Lys side-chain) and the external aldimine **2** (substrate α-amino group). Deprotonation^69,70^ of **2** results in the ylide intermediate **3** which is subsequently reprotonated from the other face to produce the external aldimine **4** with opposite configuration. In contrast, the metal-dependent enzymes, *e.g.* mandelate racemase, apparently perform a concerted reaction (Scheme 1B; **5–7**). Solvent isotope experiments show that label is incorporated into product with little incorporation into recovered substrate,^73,74^ which is consistent with a concerted mechanism. However, kinetic isotope effect measurements on mandelate racemase are consistent with a stepwise reaction and a discrete deprotonated intermediate.^75^ Finally, most cofactor-independent racemases/epimerases utilise a concerted mechanism,^5,76–78^ as illustrated by glutamate racemase (Scheme 1C; **8–10**). However, some cofactor-independent enzymes using substrates with acidic α-protons perform their reactions with a stepwise mechanism *via* a discrete enolate intermediate (*e.g.* α-methylacyl-CoA racemase^79–81^).

Enzymes which use metal ions as Lewis acids (enolase family enzymes) or are cofactor-independent are of particular interest, since they are able to perform the apparently simple 1,1-proton transfer using active site amino-acid residues and thus are model systems for understanding enzymatic reactions in general. Several of these enzymes are also important as drug targets,^6,7,20–23^ potential drug targets,^84^ or are used in biotechnological applications.^48,49,51^ This review will consider racemases/epimerases utilising deprotonation and deprotonation mechanisms, their reactivity and the strategies used to inhibit them.

## Reactivity of racemases and epimerases

### Racemisation and epimerisation reactions

On the face of it, the reaction catalysed by racemases and epimerases operating through a 1,1-proton transfer mechanism is deceptively simple, consisting of only deprotonation and deprotonation (Scheme 1). In the case of the PLP-dependent enzymes, *e.g.* alanine racemase, the active site is situated at the interface between two dimer subunits.^69^ Formation of the external aldimine between the PLP cofactor and substrate considerably enhances the acidity of the C_α_–H.^5,18,20,69^ Stabilisation of the developing negative charge in PLP-dependent enzyme reactions requires that the broken bond is perpendicular to the PLP π-system.^69,70,85^

The imine nitrogen between the amino-acid substrate and the PLP cofactor is thought to be protonated^5^ and this enhances the acidity of the C_α_–H. This effect is illustrated by chemical systems which show that the p*K*_a_ of zwitterionic glycine is 28.9 whilst the corresponding p*K*_a_ for the zwitterionic imine between glycine and acetone is 22.^86^ Model studies using the glycine aldimine of pyridoxal suggest a C_α_–H p*K*_a_ value of 11 and 17 for when the pyridoxal aromatic hydroxy group is protonated and deprotonated, respectively.^5^ These studies also show that protonation of the amino-acid carboxylate further decreases the C_α_–H p*K*_a_ value to 6 but crystal structures suggest that this does not occur during the enzyme catalytic cycle. This is contrast to the situation in cofactor-independent racemases/epimerases, where substrate carboxylate groups are held within a hydrogen-bonding network^20^ or transiently protonated during the reaction.^21^ The p*K*_a_ of the external aldimine C_α_–H in the alanine racemase reaction is estimated to be 9, which is intermediate between those for the catalytic bases, Tyr-265 and Lys-39.^69^

**Scheme 2:**
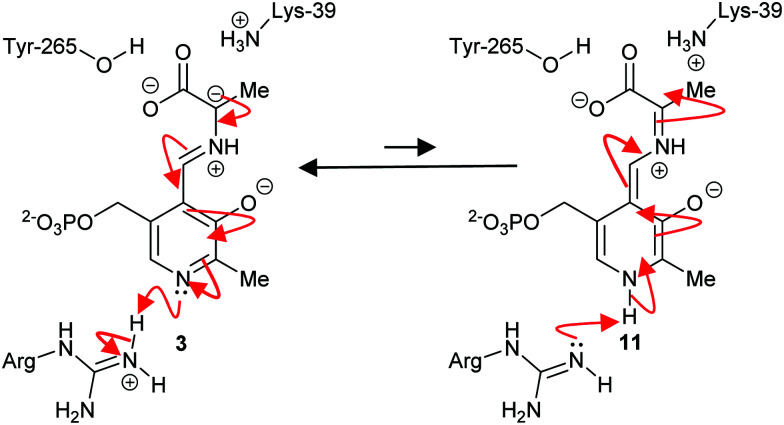
Carbanion **3** and quinoid **11** intermediates in the alanine racemase reaction.^69^

The mechanism of some PLP-dependent enzymes, *e.g.* ornithine decarboxylase, is thought to go *via* a quinoid intermediate^69,70^ resulting from protonation of the pyridoxal nitrogen by a glutamic acid residue. The equivalent residue in alanine racemase is an arginine and the pyridoxal nitrogen is not extensively protonated^69,70^ (Scheme 2). Therefore, alanine racemase is thought to catalyse its reaction *via* a carbanion **3** not a quinoid **11** intermediate.^69^ Kinetic isotope effect experiments on alanine racemase are consistent with a carbanion rather than quinoid intermediate.^70,87^

The situation is different for enolase-family racemases and epimerases and those which are cofactor-independent. The fundamental problem for these enzymes is how to deprotonate a substrate carbon acid (typical p*K*_a_ = ∼21–23^86,88,89^) using active site bases with p*K*_a_ values in the range 6–9 without the enhancement afforded by a PLP cofactor. Many racemase/epimerase substrates possess carboxylic acids (p*K*_a_ 2–5), which are deprotonated to the negatively charged carboxylate (*e.g.* substrates of amino-acid racemases/epimerases^20^ and methylmalonyl-CoA epimerase^90^). Consequently, the apparent p*K*_a_ of the C_α_–H for these substrates will be ∼29.^20,86^ This effect is illustrated by chemical systems, which show that the p*K*_a_ for the C_α_–H of glycine in water is 28.9, while the corresponding p*K*_a_ for glycine methyl ester is 21.0.^86^

Racemases and epimerases utilising a negatively charged substrate generally hold the carboxylate group within a hydrogen-bonding network or ion pair to disperse the negative charge.^20^ In some cases, the enzyme also transfers the incoming proton onto the substrate carboxylate group before it is transferred onto the C_α_ of the product (*e.g.* glutamate racemase^21^). Exceptions to this strategy are seen with methylmalonyl-CoA epimerase^90^ and mandelate racemase,^91,92^ where the carboxylate group is ligated to the active site Co^2+^ or Mg^2+^ ion which acts as a Lewis acid and diminishes the p*K*_a_ of the C_α_–H.^21^ Typically the carboxylate group is also held within a hydrogen-bonding network with active-site residues.^5^ Some racemase/epimerase substrates also contain further destabilising groups, such as ammonium groups (amino-acid racemases/epimerases),^18,20^ amide carbonyl groups (*N*-succinylamino acid racemases, dipeptide epimerases and other enolase family enzymes^18,49^) and OH (mandelate racemase,^18,91^ various sugar epimerases^18^). Both ammonium and OH groups are more easily deprotonated than the C_α_–H. Chemical models^86,93^ show that the p*K*_a_ of the C_α_–H is diminished by 9–15 units by protonation of an adjacent amine and a number of amino-acid racemases/epimerases,^20,94^ including diaminopimelate epimerase and glutamate racemase, appear to protonate the amine of the substrate during the reaction. In the case of mandelate racemase^18,91^ and *N*-succinylamino-acid racemases,^49^ the OH or amide carbonyl groups are ligated to active-site metals such as Mg^2+^ (mandelate racemase^18,91,92^) or Co^2+^, Mn^2+^ or, occasionally, Mg^2+^ (*N*-succinylamino-acid racemases^49^). The rates of proton transfer for the deprotonation and reprotonation steps are generally high, with rate constants of the order of 5 × 10^9^ to 100 × 10^9^ M^−1^ s^−1^.^86^

Recent analysis^18^ of racemase/epimerase crystal structures, obtained in the presence of ligands, suggests that the vast majority of enzymes bind the two substrate stereoisomers using ‘mirror-image packing’, that is functional groups are held within the same position with the C_α_–H on opposite sides in the different stereoisomers. In some cases, *e.g.* amino-acid racemases/epimerases,^20^ the positions of the substrate side-chain and functional groups show remarkably small differences in their positions between the stereoisomers. In other cases, *e.g.* AMACR/MCR^6,7,18,95^ which utilises substrates with large hydrophobic side-chains, the different epimers are accommodated by fixing two of the function groups (the methyl group and acyl-CoA moiety in this case) whilst the side-chain is accommodated in discrete binding sites on a hydrophobic surface at the entrance of the active site.

The active-site bases sit immediately adjacent to the C_α_–H. In the vast majority of cases, the active-site bases are located on both sides of the substrate (the so called ‘two-base enzymes’), while, in a few cases (the ‘one-base’ enzymes), a single active-site base mediates catalysis.^18,20,96^ Many racemases/epimerases are dimers, with the active site located at the dimer interface and active-site bases contributed by both subunits;^18,20,95^ binding of substrate often triggers movement of the subunits from an ‘open’ to a ‘closed’ conformation, moving the active-site bases into position and desolvating the active site.^20,94,97,98^ In some enzymes (*e.g.* glutamate racemase^21^), this conformational change triggers a change in the conformation of the deprotonating active-site base as part of the pre-activation step which results in protonation of the substrate carboxylate group. It has also been suggested that conformational changes by ‘capping domains’, which result in the closed form of the racemase, activate the enzyme for catalysis, are important, *e.g.* in mandelate racemase.^94^ In other cases, little or no conformational changes are observed in the protein upon binding of substrate and the enzyme active site is substantially desolvated in the unbound state.^20,95^

### Active-site bases

**Fig. 1:**
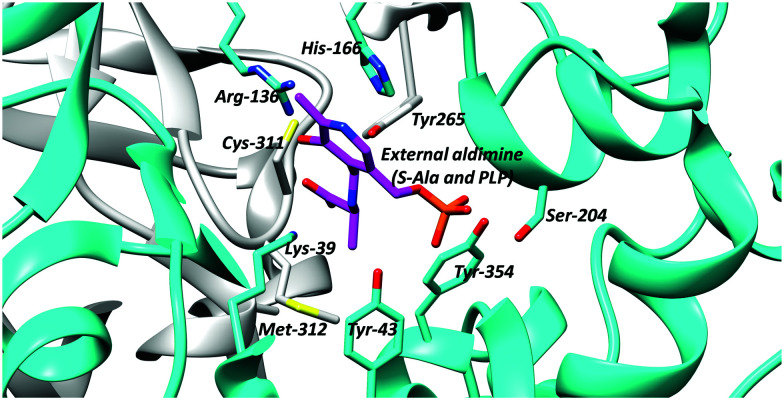
Active site residues of *B. stearothermophilus* alanine racemase showing the external aldimine (alanine conjugated to PLP) and active site bases Lys-39 and Tyr-265 (PDB: 1L6F).^99^

**Scheme 3:**
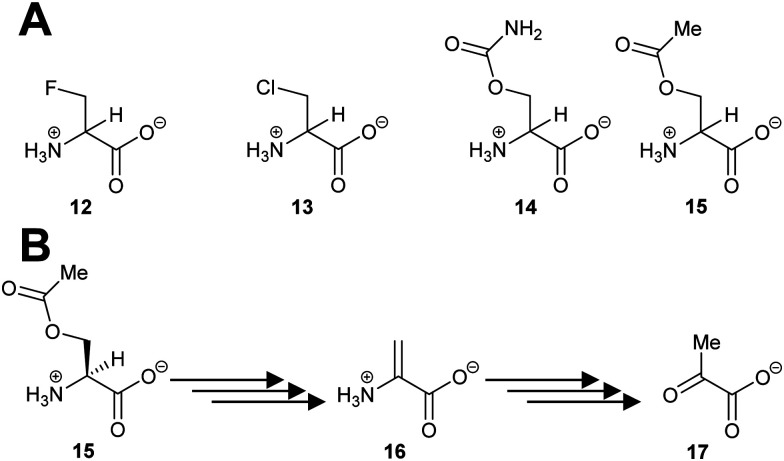
(A) Structures of eliminating inhibitors and substrates of *E. coli* alanine racemase; (B) conversion of *O*-acetyl-*S*-serine **15S** to pyruvate **17** by alanine racemase.^132^

PLP-dependent enzymes use several different active-site bases. In alanine racemase, these are generally thought to be Tyr-265 and Lys-39 (Fig. 1).^5,69,70,99^ Chemical models suggest that the p*K*_a_ of these active-site bases are increased to ∼21 (Lys) and ∼28 (Tyr), respectively, in the hydrophobic active site.^100^ This is considerably higher than the experimentally-determined C_α_–H p*K*_a_ value of 11^101^ and 9.94.^87^ Hence, deprotonation of the substrate is expected to be facile. In serine racemase, the corresponding active-site bases are Lys-57 and Ser-82^69^ and the experimentally determined external aldimine C_α_–H p*K*_a_ value is 9.26.^87^ Chemical models suggest that their active site base p*K*_a_ values will be ∼21 and 33–39,^100^ the latter being extremely high. These p*K*_a_ values will be modified by hydrogen-bonding networks within the active site, to allow deprotonation of the active-site residues and reprotonation of the carbanionic intermediate (*vide supra*, Schemes 1 and 2, **3**).

**Fig. 2:**
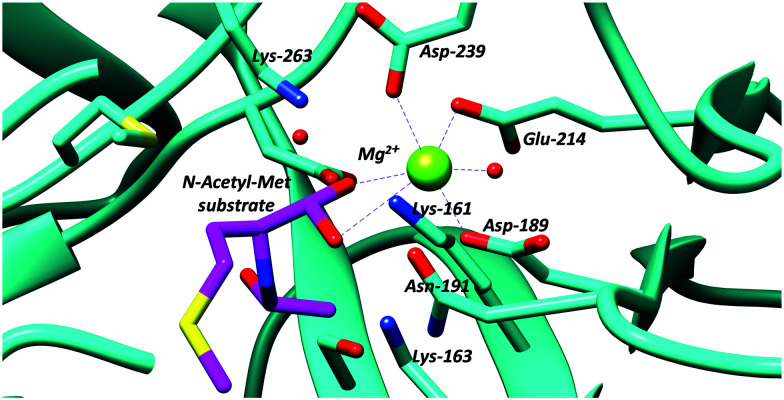
Active site residues of an *N*-acetyl-amino-acid racemase, showing binding of *N*-acetyl-methionine substrate (PDB: 4A6G).^104^

The *N*-succinylamino acid racemases and related enolase enzymes, *e.g. O*-succinylbenzoate synthase,^102,103^ utilise a pair of lysine residues as catalytic bases^49,104^ (Fig. 2). Chemical models^100^ suggest that the p*K*_a_ for these lysine residues within the active site will be ∼21. The C_α_–H p*K*_a_ for these substrates ligated to active-site metals appears not to have been calculated, though studies on other metal-dependent enzymes (mandelate racemase)^101^ suggests that this will be ∼15.

**Fig. 3:**
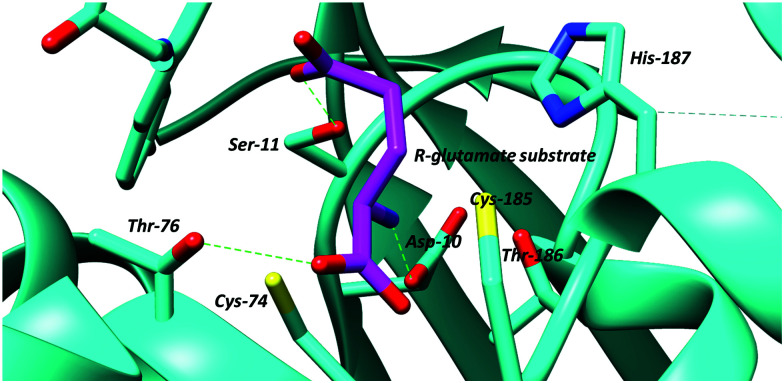
Active site of glutamate racemase from *B. subtilis* showing bound *R*-glutamate substrate and active site bases, the cysteine residues, Cys-74 and Cys-185 (PDB: 1ZUW).^105^ Hydrogen bonds are shown as green dashed lines.

Several different active-site bases are used by the cofactor-independent racemases and epimerases. In most amino-acid racemases/epimerases, both active-site bases are Cys, which act as a thiolate base/thiol acid pair, catalysing deprotonation and deprotonation,^18,20^*e.g.* Cys-74 and Cys-185 in *B. subtilis* glutamate racemase^105^ (Fig. 3). Cys is favoured as an active-site base in amino-acid racemases and epimerases because it is more easily desolvated and has a lower p*K*_a_ than Ser or Thr.^106^ Chemical models suggest that desolvation raises the p*K*_a_ of the Cys residue thiol to thiolate conversion to ∼28,^100^ matching the expected p*K*_a_ of the C_α_–H of ∼29.^20,86^ This allows deprotonation of the C_α_–H by the Cys thiolate. In contrast, the p*K*_a_ values of the active-site Cys residues acting as an acid appear to be ∼6–7 to enable protonation from the opposite side. This change in p*K*_a_ appears to be mediated by a dipole on the α-helices bearing the Cys thiol (at least in diaminopimelate epimerase^20,107^). Exceptions to this rule include aspartate/glutamate racemase from a pathogenic *E. coli* strain (*Ec*l-DER), in which one of the catalytic Cys is replaced by Thr. This enzyme catalyses irreversible conversion of *S*-Asp to *R*-Asp, which arises partly because of differences in the p*K*_a_ values of the Cys and Thr side-chains and partly because of differences in the distance between the C_α_–H and the catalytic bases on either side of the substrate.^20,108,109^ Similarly, MMP0739 aspartate/glutamate racemase from *Methanococcus maripaludis* possesses active-site Cys and Thr residues and is predicted to catalyse unidirectional enantiomerisation^42^ (the opposite catalytic base is replaced compared to the aspartate/glutamate racemase exception noted above^20^). The *H. sapiens trans*-3-hydroxy-*S*-proline epimerase^110^ also possesses an equivalent Cys-to-Thr substitution to that in MMP0739.^42^ However, biochemical analysis shows that this Cys-to-Thr substitution converts the latter enzyme from an epimerase into a dehydratase,^110^*i.e.* the enzyme catalyses elimination rather than racemisation/epimerisation (*vide infra*).

Other racemases and epimerases use a variety of active-site bases, including Cys/Cys (allantoin racemase^71^), His/Lys (mandelate racemase^71^), Glu/Glu or Asp/Asp (several different epimerases acting on sugar substrates^71^), Glu/Glu (methylmalonyl-CoA epimerase^90^), Tyr/Glu (heparin sulfate d-glucuronosyl C-5 epimerase^71^), Glu/His or Tyr/His (various sugar mutarotatases^71^), Lys/Lys (various *N*-succinylamino-acid racemases and enolase family racemases^18,49^), an Asp/His pair and Tyr (dTDP-diphosphate-4-keto-6-deoxyglucose 3,5-epimerase a.k.a. RmlC),^111^ and a Glu/His pair and Asp (AMACR and MCR^71,81,95,112^). *N*-Acetylmannosamine-6-phosphate 2-epimerase appears to be an exception to this rule, as only one active site base/acid (Lys) has been identified.^18,96^

The active sites of these other racemases and epimerases also exclude bulk solvent.^95^ Chemical models^100^ again suggest that the p*K*_a_ of these active-site bases are correspondingly increased to ∼29 (His), ∼21 (Lys), ∼22 (Asp and Glu), and ∼28 (Tyr), again matching approximately the expected p*K*_a_ values of the substrate C_α_–H. Each of these bases participates in a hydrogen-bonding network with other active site residues and, in some cases, active-site ordered waters.

**Fig. 4:**
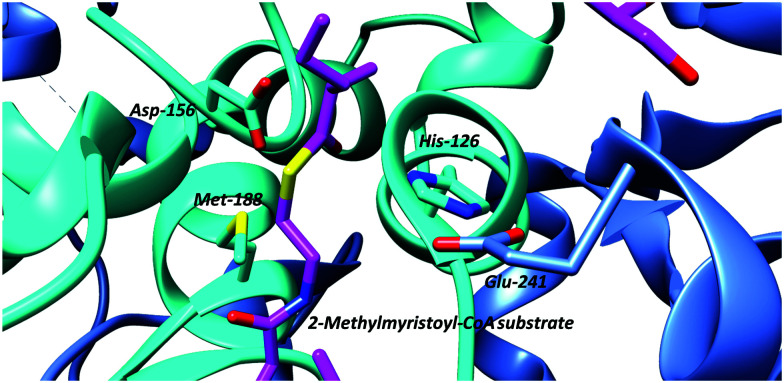
Active-site arrangement of α-methylacyl-CoA racemase (MCR) from *M. tuberculosis* showing binding of binding of 2-methyltetradecanoyl-CoA substrate (PDB: 2GCI).^95^ Active site bases include Asp-156 and the His-126/Glu-241 pair, with Glu-241 contributed by the second monomer subunit. The His-126/Glu-241 pair removes the α-proton of the *S*-2-methylacyl-CoA substrate whilst Asp-156 protonates the enolate intermediate.^6,7,81,95^ The roles of these residues are reversed for the *R*-2-methylacyl-CoA substrate. Met-188 stabilises formation of the enolate intermediate.

An often-overlooked consideration in the catalytic mechanism is the hydrogen bonding between the electron-deficient C_α_–H (which are activated by adjacent carbonyl groups) and active-site bases. This is of relevance for all proteins, since all protein amino-acid residues are capable of forming such bonds.^113^ These hydrogen bonds tend to be moderately weak (8 to 10.6 kJ mol^−1^ when bonding to water compared to 18.9 kJ mol^−1^ for an ‘typical’ intra-molecular bond^114^). In addition, amino-acids and other racemase/epimerase substrates will also be able to form such bonds. The case of the C_α_–H/His/Glu hydrogen bond in AMACR/MCR is particularly interesting in this regard (Fig. 4), as the hydrogen bond resembles that in the catalytic triad of chymotrypsin and related hydrolytic enzymes which has been studied in detail.^115^

### Concerted *versus* stepwise reactions

The PLP-dependent enzymes have been extensively studied and a series of mechanistic and computational studies show the presence of a carbanionic intermediate (*vide supra*, Schemes 1 and 2, **3**),^5,50,69,70,101^ indicating a step-wise reaction. Kinetic isotope effect studies on alanine racemase are also consistent with a carbanionic intermediate.^87^ Alanine racemase catalyses C_α_–H exchange but the stereochemical course of this reaction was not determined,^116^ although non-stereoselective incorporation of label into substrate is expected because of the stability of the carbanionic intermediate.

Studies investigating isotopic incorporation from solvent into substrates have been particularly informative about the concertedness of mechanism in other enzymes. For the majority of enolase family and cofactor-independent racemases and epimerases, isotopic incorporation is observed into the product but very little incorporation is observed into the substrate, *e.g.* glutamate racemase,^78^ proline racemase,^77^ mandelate racemase,^74^ 2-methylmalonyl-CoA epimerase^73^ and a racemase mediating post-translational modification of peptides.^117^ This is consistent with a concerted reaction. Monitoring the progress of the reaction by these enzymes in isotopically labelled solvent using circular dichroism typically results in an over-shoot of the equilibrium position, *e.g.* as has been observed for mandelate racemase.^74^ This results from isotopic incorporation into product only with a significant kinetic deuterium isotope effect affecting the reverse reaction. These results further support a mechanism in which two-base enzymes catalyse a microscopic enantiomerisation reaction, with asynchronously concerted deprotonation and reprotonation.^76^ Such a mechanism minimises the formation of a highly unstable doubly deprotonated intermediate and hence partly overcomes the effect of destabilising groups adjacent to the C_α_–H (*i.e.* the carboxylate).

In contrast to the above is the observation that incubation of substrates with AMACR in ^2^H_2_O results in a near 1 : 1 incorporation of deuterium into substrate and product. This has been interpreted as formation of a discrete deprotonated intermediate followed by deuteration from either side.^79,80^ Analysis of the crystal structure of the *M. tuberculosis* homologue, MCR, shows catalytic residues on both sides of the substrate (the His-126/Glu-241 pair and Asp-156; Fig. 4) and are consistent with the formation of an enolate intermediate.^81,95,112^ Thus, AMACR and MCR fundamentally differ in their mechanisms from most other cofactor-independent racemases and epimerases, in that they catalyse microscopic racemisation rather than epimerisation.^8,79,80^ Incorporation of deuterium from solvent is also catalysed by hydantoin racemase *via* an enolate intermediate^118^ and is expected to be non-stereoselective but this has not yet been verified.

The above results can be rationalised based on the p*K*_a_ values for the deprotonation of the substrate. The p*K*_a_ of C_α_–H for a thioester is 21,^86,88^ while the p*K*_a_ values for C_α_–H for amino-acid zwitterions is 29,^20,86^ for simple carboxylates is 33 and for simple amides 28.4.^86^ Therefore, concerted reactions occur with substrates containing relatively unactivated C_α_–H (high p*K*_a_ values), with consequent asymmetrical isotopic incorporation. This explains the behaviour of peptide epimerases,^117^ which are observed to undergo concerted reactions. These peptide substrates have p*K*_a_ values of ∼26–31 for C_α_–H, although these values are dependent on both *N*- and *C*-substituents and the protonation status of amine groups.^86^ This model also allows prediction of enzymatic behaviour for uncharacterised racemases/epimerases, *e.g.* hydantoin racemase,^118^ based on p*K*_a_ values for C_α_–H. The proposed model also casts doubt on the use of isotopic labelling studies to differentiate between ‘two-base’ and ‘one-base’ enzymes (reviewed in ref. 92). It has previously been proposed that near-symmetrical isotopic incorporation into substrate and product is indicative of ‘internal return’, *i.e.* a ‘one-base’ mechanism. The results on AMACR^79,80^ (reviewed in ref. 6 and 7) show that this behaviour is also observed with ‘two-base’ enzymes with activated C_α_–H, as it is known that AMACR/MCR possesses appropriate active-site bases on both sides of the substrate.

### Elimination reactions

Several racemases/epimerases catalyse elimination reactions, in addition to racemisation/epimerisation. With the exception of the ‘mutant’ *H. sapiens trans*-3-hydroxy-*S*-proline epimerase containing a Cys-to-Thr substitution noted above^110^ giving rise to dehydratase activity, and the *Labrenzia aggregata cis*-3-hydroxy-*S*-proline racemase/dehydratase (IAM 12614)^119^ (*vide infra*), all of the known elimination reactions take place with unnatural substrates. The vast majority of these unnatural substrates are halogen derivatives,^20,47,63,82,120–126^ with only a few exceptions.^63,119,124,127,128^ The deprotonation step in the elimination reaction is highly similar to that described for racemisation/epimerisation (*vide supra*).

Several PLP-dependent racemases catalyse elimination reactions.^129–133^ The classic example is alanine racemase (Scheme 3) which β-eliminates halogens from 3-fluoroalanine **12** and 3-chloroalanine **13**.^132^*O*-Carbamoyl-*R*-serine **14R** and *O*-acetyl-*R*-serine **15R** act as irreversible inhibitors whilst *O*-carbamoyl-S-serine **14S** and *O*-acetyl-*S*-serine **15S** are reversible competitive inhibitors.^132^ 3-Fluoroalanine **12** is a potent inactivator of alanine racemase. 3-Chloroalanine **13** and *O*-carbamoyl-*S*-serine **14** and *O*-acetyl-*S*-serine **15** also act as substrates. These substrates result in the formation of 2-aminoacrylate **16**, which tautomerises to pyruvate **17** with a partition coefficient of between 790 and 920 to 1 (catalytic conversion/inactivation).

There have also been several studies on the elimination reaction catalysed by *H. sapiens* serine racemase.^129–131,133^ The wild-type enzyme has a *ca.* 4-fold preference for β-elimination over racemisation of *S*-serine.^129,131^ Other substrates can also undergo β-elimination, including *S*-serine-*O*-sulfate and *S-threo*-hydroxyaspartate.^131^ The enzyme is allosterically activated by divalent metal ions (with Mn^2+^ being the strongest) and ATP,^129,133^ and activity is potentiated by halide anions.^130^ The elimination reaction catalysed by serine racemase is thought to control levels of *R*-serine in neurons^133^ and, hence, modulate the activity of NMDA receptors;^129,131,133^ over-activation of the NMDA receptor has been shown to result in neuronal cell death.^133^ This is, however, at the expense of producing highly electrophilic 2-aminoacrylate **16**.^129,131,133^

**Scheme 4:**
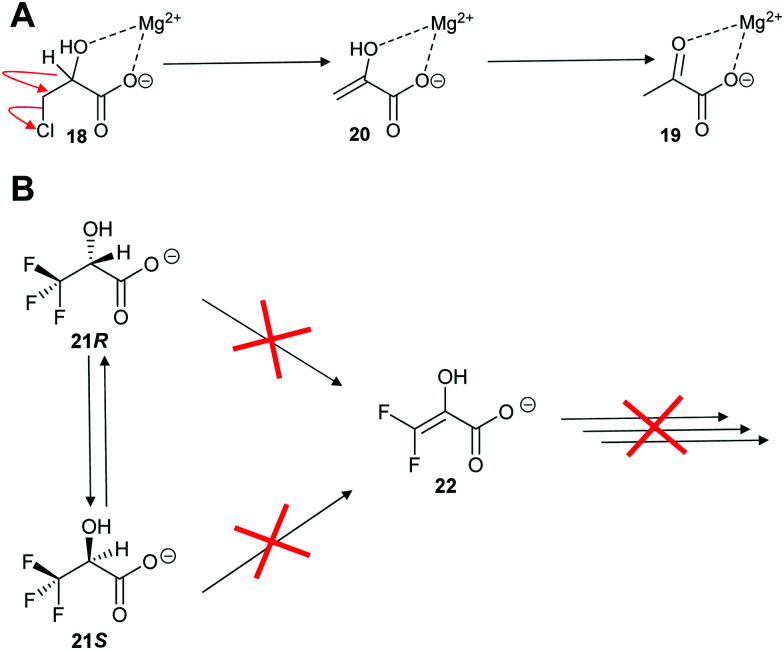
Reaction of halogen substrates with *P. putida* mandelate racemase.^91,126^ (A) Elimination of chlorine from 3-chlorolactate **18**;^126^ (B) expected elimination of 3,3,3-trifluorolactate **21** to give **22**.^91^

Enolase family enzymes, such as *P. putida* mandelate racemase^126^ and *L. aggregata cis*-3-hydroxy-*S*-proline racemase/dehydratase (IAM 12614),^119^ are also able to catalyse elimination reactions. Mandelate racemase was able to catalyse elimination of chlorine from 3-chlorolactate **18** to give pyruvate **19** (Scheme 4A).^126^ The mechanistic details of the reaction was not determined but it is assumed to occur by E2 *anti*-elimination to give the enol **20** followed by tautomerisation.^126^ However, the possibility of a E1cb-type mechanism *via* an enediolate type intermediate cannot be discounted. The elimination of chlorine from 3-chlorolactate **18** by mandelate racemase is reminiscent of the elimination of HCl from 3-chloroalanine **13** by glutamate racemase, which also gives pyruvate **17** as a product (*vide infra*, Scheme 12).^134^ This result contrasts with the earlier observation on *P. putida* mandelate racemase with 3,3,3-trifluorolactate **21**, which undergoes racemisation. β-Elimination to give **22** is not observed (Scheme 4B).^91^

**Scheme 5:**
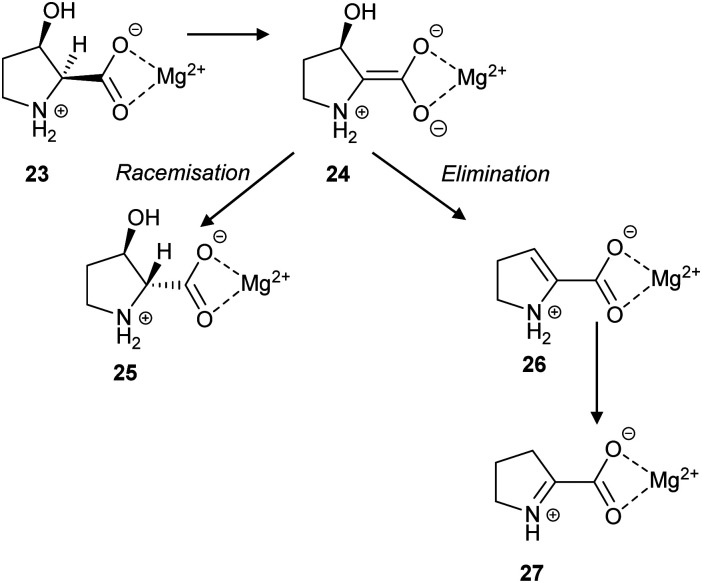
The racemisation and elimination reactions catalysed by *cis*-3-hydroxy-*S*-proline racemase/dehydratase.^119^

*L. aggregata cis*-3-hydroxy-*S*-proline racemase/dehydratase (IAM 12614)^119^ catalyses both racemisation and β-elimination reactions with its substrate **23**, in a 3 to 2 ratio (Scheme 5). The β-elimination reaction is proposed to go *via* an enediolate intermediate **24**, although it may be a more concerted E2-like reaction. The *cis* substrate allows for *anti*-elimination of the hydroxy group to give the enamine product **26**, which subsequently tautomerises to Δ-pyrroline-2-carboxylate **27** (Scheme 5). Alternatively, epimerisation to give **25** can occur. It is notable that **27** is a known inhibitor of *T. cruzi* proline racemase.^135^

**Scheme 6:**
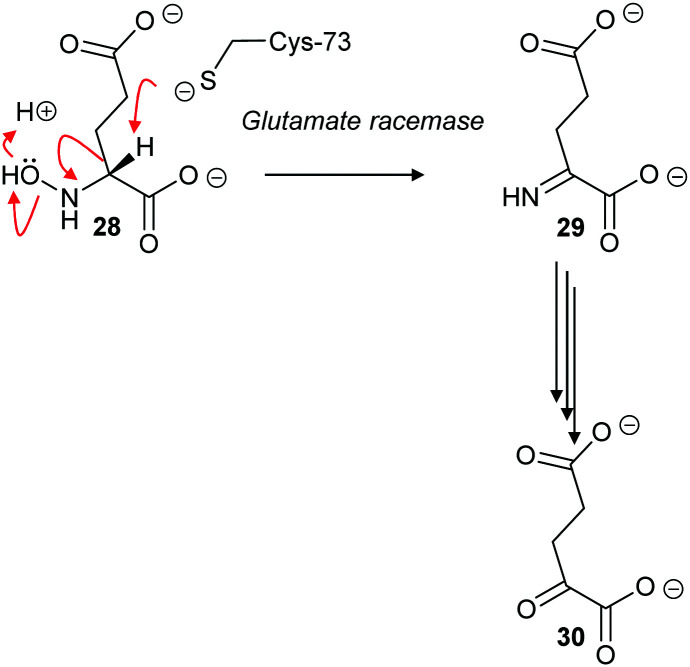
Elimination of *N*-hydroxy-*R*-glutamate **28** by an E2 mechanism followed by hydrolysis of imine **29** to form 2-oxoglutarate **30**.^128^

The cofactor-independent enzymes diaminopimelate epimerase^124^ and glutamate racemase^128^ are able to eliminate *N*-hydroxy substrates. In the case of glutamate racemase, deprotonation of substrate **28** results in elimination of hydroxide or water with formation of imine **29**, which is hydrolysed to 2-oxoglutarate **30** (Scheme 6).

**Scheme 7:**
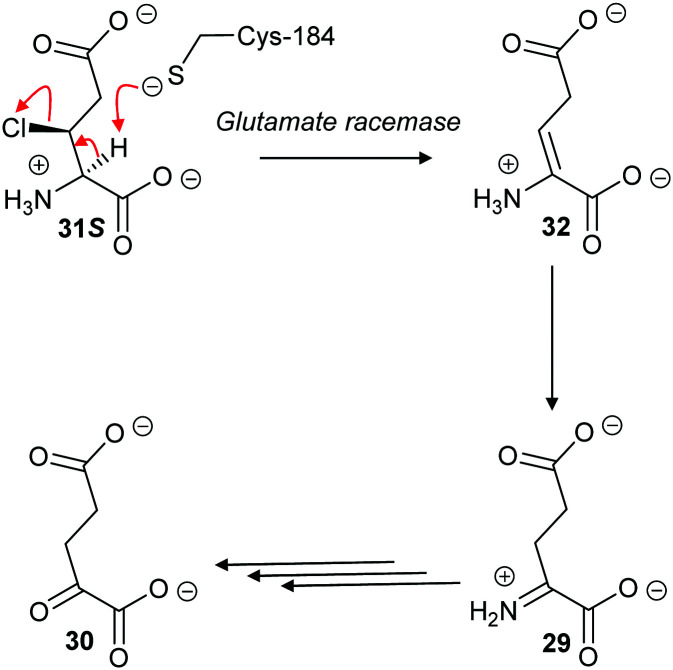
β-Elimination of 2*S*,3*S*-3-chloroglutamate **31S** by *Lactobacillus* glutamate racemase to give enamine **32**. Tautomerisation to imine **29** followed by hydrolysis gives the resulting 2-oxoglutarate **30**.^82^

With aliphatic substrates containing β-fluorine or β-chlorine, the presence of the halogen increases the acidity of the C_α_–H,^120,136^ and, hence, these elimination substrates tend to be converted with somewhat higher efficiency than their racemisation/epimerisation equivalents.^120^ With diaminopimelate epimerase,^121^ only stereoisomers allowing an antiperiplanar conformation between the C_α_–H and the fluorine underwent elimination, with substrates not allowing an antiperiplanar conformation undergoing epimerisation instead. Similarly, mutant glutamate racemases (in which the active-site Cys bases were mutated to Ser) eliminated either 2*R*,3*R*- or 2*S*,3*S*-3-chloroglutamate stereoisomers **31R** and **31S** with *anti*-elimination (Scheme 7), depending on which active site Cys residue was still present.^82^ The resulting enamine **32** tautomerises to imine **29**, which is hydrolysed to 2-oxoglutarate **30**. These results are consistent with a substantially concerted (E2) mechanism.^137^

**Scheme 8:**
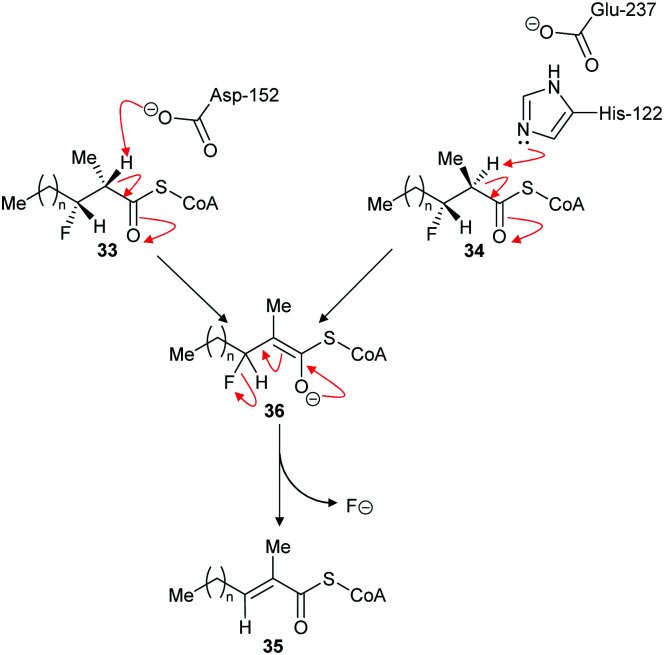
Elimination of fluoride from substrates by α-methylacyl-CoA racemase.^120^ For substrates **33** and **34**, *n* = 6. For inhibitors (**34** and its epimer with opposite C2 and C3 configuration) tested on native rat enzyme reported not to eliminate,^136^*n* = 12. Enzyme catalytic residue numbers are those for human AMACR.^6,7^

The above results contrast with those observed with AMACR, in which epimeric substrates **33** and **34** were eliminated to the same product **35**,^120^ consistent with an E1cb mechanism through the enolate intermediate **36** (Scheme 8).^137^ These results are inconsistent with an E2-elimination because the substrate requires a conformation in which the α-H and the fluorine are *anti*-; epimer **33** can adopt such a conformation but epimer **34** cannot. Interestingly, compounds closely related to **33** and **34** were synthesised^136^ and tested as inhibitors of native rat AMACR and no elimination of fluoride was observed.^136^ These inhibitors^136^ had the same configuration as **34** (and its epimer with opposite C2 and C3 configurations) but, in view of the subsequent report,^120^ this is a surprising observation.

However, this is not the only example where an expected elimination reaction did not take place. Nagar *et al.* investigated trifluorolactate (2-hydroxy-3,3,3-trifluoropropanoate) **9R** and **9S** as substrates for mandelate racemase (*vide supra*, Scheme 4).^91^ Kinetic analysis showed that *K*_m_ values for trifluorolactate **9** were unexpectedly similar to the natural substrate, mandelate (1.2–1.74 mM and 1.0–1.2 mM, respectively) and lower than the predicted *K*_m_ value of ∼10 mM. In contrast, *k*_cat_ values were reduced by ∼318-fold, with *k*_cat_/*K*_m_ reduced by ∼430-fold. ^19^F NMR analysis showed that no fluoride was eliminated during the reaction and, hence, **10** was not formed.^91^ The lack of a β-elimination reaction is unexpected, because the trifluorolactate **9** is able to take up the required *anti*-conformation for an E2 reaction when in a staggered conformation. Clearly, the mandelate racemase-catalysed racemisation of trifluorolactate is faster than the elimination reaction. The reasons for this are unclear but it could be related to the presence of multiple fluorine atoms within the substrate.^138^ If loss of fluoride is asynchronous with abstraction of the C_α_–H, this will result in generation of a positive charge on the β-carbon. The presence of two additional fluorine atoms will destabilise formation of this transition state. However, it is notable that *E. coli* dipeptide epimerase (YcjG) eliminates fluoride from *S*-alanyl-*R*,*S*-difluoroalanine in preference to epimerisation,^125^ suggesting that other factors are also at play such as the extent of δ+ charge stabilisation in the transition state.

## Methods for determining racemase and epimerase activity

Racemases and epimerases are simple enzymes, in the sense that they only have one substrate and product (a uni-uni reaction), which is a characteristic they share with other isomerases. A consequence of racemases and epimerases accepting both stereochemical configurations of their substrates is that their reaction will, in most cases, be readily reversible and *k*_cat_/*K*_m_ values are likely to be similar for the reactions in both directions (which is required by the Haldane relationship^79,80,139^ for an equilibrium constant of ∼1). Hence the rate for the reverse reaction when determining initial rates is likely to be significant and this must be corrected for in any kinetic study, such as the determination of inhibitor potency.

Several different assays exist for measuring racemase/epimerase activity (Table 1). One approach is to measure rates at very early time points where the reverse reaction will have less impact. Typically, the enzyme reaction is followed using techniques such as optical rotation^46,140–145^ or circular dichroism,^23,91,107,146–150^ allowing a time-course to be determined. These assays are ideal, in that they allow much more accurate determination of initial rates,^151^ although correction for the reverse reaction should still be performed. Indeed, circular dichroism is by far the most common method of detecting enzymatic activity (Table 1), although it is noted that these are kinetic studies designed to measure *K*_m_, *k*_cat_ and *k*_cat_/*K*_m_ (*vide infra*).

**Table 1:** Assays and catalytic parameters for racemases and epimerases

Enzyme	Type	Substrate	Reaction	Assay type	*K*_m_ (μM)	*k*_cat_ (s^−1^)	*k*_cat_/*K*_m_^a^ (M^−1^ s^−1^)
*Baccilus psychrosccharolyticus* alanine racemase^177^	PLP-dependent	*S*-Alanine	Racemisation	Polarimetry	17 900	715^b^	39 944
*Baccilus psychrosccharolyticus* alanine racemase^177^	PLP-dependent	*R*-Alanine	Racemisation	Polarimetry	12 200	1417^b^	116 147
*Corbicula japonica* Alanine racemase^178^	PLP-dependent	*S*-Alanine	Racemisation	Coupled enzyme (d-amino acid oxidase)	22 600	430^b^	19 026
*Corbicula japonica* Alanine racemase^178^	PLP-dependent	*R*-Alanine	Racemisation	Coupled enzyme (NAD^+^-dependent)	9200	196^b^	21 304
*Tolypocladium inflatum* alanine racemase^179^	PLP-dependent	*S*-Alanine	Racemisation	Coupled enzyme (d-amino acid oxidase)	7000	3.8	543
*Tolypocladium inflatum* alanine racemase^179^	PLP-dependent	*R*-Alanine	Racemisation	Coupled enzyme (NAD^+^-dependent)	2700	1.51	559
*E. coli* alanine racemase^134^	PLP-dependent	*S*-Alanine	Racemisation	Coupled enzyme (NAD^+^-dependent)	340 ± 10	170 ± 2	500 000
*B. subtilis* alanine racemase^134^	PLP-dependent	*S*-Alanine	Racemisation	Coupled enzyme (NAD^+^-dependent)	5900 ± 900	1190 ± 70	201 695
*M. tuberculosis* alanine racemase^134^	PLP-dependent	*S*-Alanine	Racemisation	Coupled enzyme (NAD^+^-dependent)	3700 ± 600	37 ± 2	10 000
*L. otakiensis* isoleucine 2-epimerase^180^	PLP-dependent	2*S*-Isoleucine	Epimerisation	UPLC	5000 ± 80	502 ± 16.2	100 400
*L. otakiensis* isoleucine 2-epimerase^180^	PLP-dependent	2*R-Allo*-Isoleucine	Epimerisation	UPLC	13 200 ± 644	939 ± 26.8	71 136
*H. sapiens* serine racemase^131^	PLP-dependent	*S*-Serine	Racemisation	Coupled enzyme (d-amino acid oxidase)	7800 ± 700	0.205 ± 0.007	26.3
*H. sapiens* serine racemase^131^	PLP-dependent	*S*-Serine	Elimination	Coupled enzyme (NADH-dependent)	10 000 ± 600	0.97 ± 0.028	97.0
*H. sapiens* serine racemase^131^	PLP-dependent	*S*-Serine-*O*-sulfate	Elimination	Coupled enzyme (NADH-dependent)	1200 ± 100	12.06 ± 0.16	10 050
*H. sapiens* serine racemase^131^	PLP-dependent	*S-Threo*-3-hydroxyaspartate	Elimination	Coupled enzyme (NADH-dependent)	2500 ± 300	23.33 ± 0.266	9332
*P. putida* mandelate racemase^91^	Enolase	*S*-Mandelate	Racemisation	Circular dichroism	1000 ± 100	637 ± 31	637 000
*P. putida* mandelate racemase^91^	Enolase	*R*-Mandelate	Racemisation	Circular dichroism	1200 ± 200	792 ± 19	660 000
*P. putida* mandelate racemase^91^	Enolase	*S*-Trifluorolactate	Racemisation	Circular dichroism	1740 ± 80	2.5 ± 0.3	1437
*P. putida* mandelate racemase^91^	Enolase	*R*-Trifluorolactate	Racemisation	Circular dichroism	1200 ± 200	2.0 ± 0.2	1667
*Amycolatopsis* sp. Ts-1- 60 *N*-acyl amino acid racemase^104^	Enolase	*N*-Acetyl-*S*-Methionine	Racemisation	HPLC	18 000	20	1111
*Amycolatopsis* sp. Ts-1- 60 *N*-acyl amino acid racemase^104^	Enolase	*N*-Acetyl-*R*-Methionine	Racemisation	HPLC	40 000	14	350
*N*-acylamino acid racemase^181^	Enolase	*N*-Acetyl-*R*-methionine	Racemisation	HPLC	11 470 ± 1360	0.809 ± 0.027	70.6
*N*-acylamino acid racemase^181^	Enolase	*N*-Acetyl-*R*-methionine	Racemisation	Coupled enzyme (acylase, d-amino acid oxidase), colorimetric	23 410 ± 2120	2.55 ± 0.09	108.9
LvNSAR/OSBS^182^	Enolase	*N*-Succinyl-*R*-phenylglycine	Racemisation	Polarimetry	2700 ± 540	2.2 ± 0.2	815
RcNSAR/OSBS^182^	Enolase	*N*-Succinyl-*R*-phenylglycine	Racemisation	Polarimetry	1800 ± 230	15 ± 4	8333
AmedNSAR^182^	Enolase	*N*-Succinyl-*R*-phenylglycine	Racemisation	Polarimetry	2800 ± 550	74 ± 7	26 429
ExiOSBS^182,183^	Enolase	*N*-Succinyl-*S*-phenylglycine	Racemisation	Polarimetry	1700 ± 500	0.07 ± 0.006	41.2
GkNSAR/OSBS^182,184^	Enolase	*N*-Succinyl-*S*-phenylalanine	Racemisation	Polarimetry	800 ± 200	19 ± 1	23 750
AmyNSAR^182,185^	Enolase	*N*-Succinyl-*S*-phenylglycine	Racemisation	Polarimetry	1000 ± 10	42 ± 2	42 000
DrNSAR^182,186^	Enolase	*N*-Succinyl-*S*-phenylglycine	Racemisation	Polarimetry	1400 ± 200	520 ± 30	371 429
*M. aeruginosa* Aspartate racemase (McyF)^187^	Cofactor-independent	*S*-Aspartate	Racemisation	Coupled enzyme (d-amino acid oxidase)	22 900 ± 2100	42.5 ± 1.3	1856
*M. tuberculosis* diaminopimelate epimerase^167^	Cofactor-independent	*S*,*S*-Diaminopimelate	α-^3^H for α-^1^H exchange	Isotopic wash-out from ^3^H-labelled substrate	166	0.1465	882.5
*B. subtilis* glutamate racemase^166^	Cofactor-independent	*S*-Glutamate	Racemisation	Circular dichroism	14 000 ± 1000	42 ± 2	3000
*B. subtilis* glutamate racemase^166^	Cofactor-independent	*R*-Glutamate	Racemisation	Circular dichroism	1240 ± 80	4.72 ± 0.09	3806
*F. nucleatum* glutamate racemase^166^	Cofactor-independent	*S*-Glutamate	Racemisation	Circular dichroism	1040 ± 70	17.4 ± 0.8	16 730
*F. nucleatum* glutamate racemase^166^	Cofactor-independent	*R*-Glutamate	Racemisation	Circular dichroism	1700 ± 100	26 ± 1	15 294
*C. sticklandii* proline racemase^188^	Cofactor-independent	*S*-Proline	Racemisation	Circular dichroism	5700 ± 500	97 ± 5	17 018
*H. sapiens* UDP-*N*-acetylglucosamine 2-epimerase^44^	Cofactor-independent	UDP-*N*-acetylglucosamine	Epimerisation	Coupled enzyme (NADH)	33.1 ± 4.2	11.8 ± 2.0	356 495
*C. sticklandii* proline racemase^188^	Cofactor-independent	*R*-Proline	Racemisation	Circular dichroism	3900 ± 400	51 ± 1	13 077
*B. subtilis* RacX^168^	Cofactor-independent	*S*-Lysine	Racemisation	HPLC	27 900 ± 2670	0.0013 ± 0.000083	0.047
*Streptomyces O*-ureidoserine racemase^46^	Cofactor-independent	*S-O*-Ureidoserine	Racemisation	Circular dichroism	12 000	475	39 583
*Streptomyces O*-ureidoserine racemase^46^	Cofactor-independent	*R-O*-Ureidoserine	Racemisation	Circular dichroism	32 000	1450	45 312
*E. coli* YgeA^168^	Cofactor-independent	*S*-Homoserine	Racemisation	HPLC	171 000 ± 21 100	0.130 ± 0.001	0.76
*E. coli* YgeA^168^	Cofactor-independent	*R*-Homoserine	Racemisation	HPLC	25 100 ± 4170	0.019 ± 0.0011	0.76
*Agelenopsis aperta* peptide epimerase^117^	Cofactor-independent	*N*-Acetyl-Gly-Leu-*S*-Ser-Phe-Ala	Racemisation	HPLC	8000 ± 1400	0.076 ± 0.005	9.5
*Agelenopsis aperta* peptide epimerase^117^	Cofactor-independent	*N*-Acetyl-Gly-Leu-*R*-Ser-Phe-Ala	Racemisation	HPLC	1100 ± 300	0.0058 ± 0.0005	5.3
*M. tuberculosis* AMACR^146^	Cofactor-independent	*S*-Ibuprofenoyl-CoA	Racemisation	Circular dichroism	86 ± 6	450 ± 14	5 232 558
*M. tuberculosis* AMACR^146^	Cofactor-independent	*R*-Ibuprofenoyl-CoA	Racemisation	Circular dichroism	48 ± 5	291 ± 30	6 062 500
*H. sapiens* AMACR^152^	Cofactor-independent	Pristanoyl-CoA	α-^3^H for α-^1^H exchange	Isotopic wash-out from ^3^H-labelled substrate	85.6 ± 17.1	0.08855 ± 0.006	1034
*H. sapiens* AMACR^127^	Cofactor-independent	2*R*,*S*-3-(2,4-Dinitrophenoxy)-2-methylpropanoyl-CoA	Elimination	Direct colorimetric	56 ± 5.9	0.088	1571

a *k*_cat_/*K*_m_ values are calculated from the reported the *k*_cat_ and *K*_m_ values given in the paper.

b *k*_cat_ values are calculated from reported *V*_max_ values in μmol min^−1^ mg^−1^ and reported molecular weights of 42 500^177^ and 41 200 Da,^178^ respectively.

A second alternative when analysing substrates undergoing racemisation or epimerisation is to use HPLC of a diastereoisomeric substrate/product mixture at a fixed time point,^37,136^ although the differences in energies between diastereoisomers means that these substrates may behave differently from natural enantiomeric substrates. Alternatively, a product containing one chiral centre can be derivatised using a chiral reagent and analysed by HPLC, GC or NMR.^8,79,80^ The latter approach is time-consuming, as several time-points for each reaction should be analysed and can be technically challenging, especially when working with the low amounts of product typically obtained from enzymatic reactions. Chiral HPLC is an option for separating enantiomeric substrates, although there appear to be no examples of this having been used.

A second approach is to measure exchange of the α-proton with isotopically labelled substrates^82,95,152–154^ or solvent,^8,73,78–80,107^ measuring reaction extent by scintillation counting, mass spectrometry or NMR. Such approaches will introduce significant kinetic isotope effects^155–158^ and deprotonation and reprotonation rates will be markedly different from each other although the extent of this will depend on levels of conversion of substrate and whether the transition states are early or late.^107^ Consequently, careful design of experiments is needed, especially where precise rate measurements are required. These approaches are often used in mechanistic studies where isotopic distribution in substrate and product is measured (*vide supra*).

**Scheme 9:**
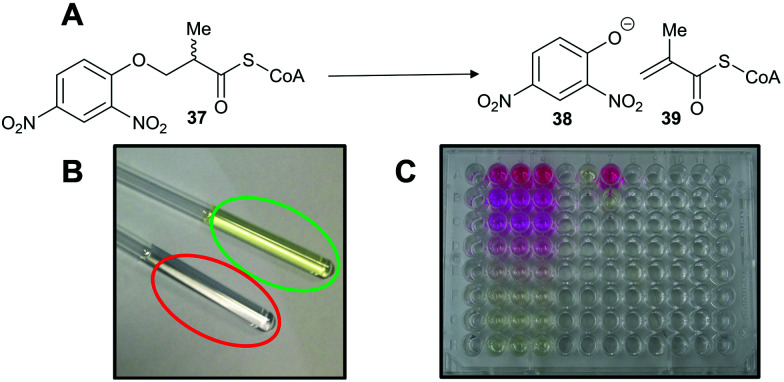
Colorimetric assay for α-methylacyl-CoA racemase (AMACR) based on elimination of 2,4-dinitrophenolate **38** from the acyl-CoA substrate **37**.^127^ (A) Reaction catalysed by AMACR; (B) assay samples showing reaction with heat-inactivated enzyme (red circle) and active enzyme (green circle) showing absorbance at 354 nm;^127^ (C) measurement of dose–response curve for Rose Bengal (a known inhibitor of AMACR^127,152^) using the colorimetric assay. Schemes 9B and C have been reproduced from Yevglevskis *et al.*, 2017^127^ with permission from the Royal Society of Chemistry.

A third approach is to make the enzymatic reaction irreversible. This can be achieved using an irreversible coupled enzyme to remove the reaction product.^107,159,160^ There are a number of examples of the use of coupling enzymes in kinetic studies determining *K*_m_ and *k*_cat_ values (Table 1). The most common coupling enzymes used are d-amino acid oxidase and NAD-dependent oxidoreductases. Coupled enzyme assays are the second most common method of assessing enzymatic activity. Similarly, an unnatural substrate which undergoes an irreversible elimination reaction^82,83,120,127,161^ can also be used. Typical examples of eliminated groups include water (from amino-acid hydroxamate derivatives^83,162^), bromide,^122,123^ chloride^47,82,124^ and fluoride^120,121,124,125,161^ as described above. The products from these elimination reactions often need to be assayed using coupling enzymes^83,121^ or low-throughput spectroscopic techniques such as NMR.^120,121,161^ Attempts to use fluoride sensors to measure enzymatic activity with substrates eliminating fluorine has met with limited success.^161^ A notable example of this approach is the elimination reaction of an unnatural acyl-CoA substrate **37** by AMACR to give 2,4-dinitrophenoxide **38** and acyl-CoA **39** (Scheme 9);^127^ this assay was used in a high-throughput screening campaign of 20 387 compounds which identified novel pyrazoloquinolines and pyrazolopyrimidines as inhibitors^163^ and also in the first extensive inhibitor structure–activity relationship studies on any racemase/epimerase (*vide infra*).^164,165^

## Catalytic efficiency of racemases and epimerases

Kinetic parameters for racemases/epimerases can vary quite widely (Table 1). *K*_m_ values for amino-acid racemases tend to be in the low mM range, although there are several examples where much higher *K*_m_ values have been measured. Typical examples include *Fusobacterium nucleatum* and *B. subtilis* glutamate racemase, which have *K*_m_ values of 1.04 and 1.07 mM and 14 and 1.24 mM, respectively.^166^ In contrast, *O*-ureidoserine racemase which has reported *K*_m_ values^46^ of 12 and 32 mM for *S*- and *R-O*-ureidoserine, respectively. In contrast, the *K*_m_ for *S*,*S*-diaminopimelate (2,6-diaminoheptanedioic acid) for *M. tuberculosis* diaminopimelate epimerase is only 166 μM,^167^ significantly lower than the *K*_m_ values for other amino-acid racemases/epimerases. The relatively high *K*_m_ values for most amino-acid racemases/epimerases are undoubtedly a consequence of these enzymes converting small and relatively unfunctionalised substrates. The same trend is observed for mandelate racemase, which has *K*_m_ values of 1.0 and 1.2 mM for *S*- and *R*-mandelate (2-hydroxyphenylacetate), respectively.^91^ Racemases/epimerases with larger substrates tend to have lower *K*_m_ values, as there is more opportunity for binding interactions. For example, human AMACR has a *K*_m_ value of ∼86 μM for pristanoyl-CoA,^152^ while *K*_m_ values for *S*- and *R*-ibuprofenoyl-CoA are 86 and 48 μM for the *M. tuberculosis* homologue.^146^ These lower *K*_m_ values are generally accompanied by lower *k*_cat_ values (Table 1).

Catalytic efficiency is quantified using *k*_cat_/*K*_m_ values (Table 1). Again, these can vary quite widely but many racemases/epimerases have relatively modest efficiencies. For example, *O*-ureidoserine racemase is quite efficient, with reported *k*_cat_/*K*_m_ values^46^ of 39 583 and 45 312 M^−1^ s^−1^. Similarly, *k*_cat_/*K*_m_ is reported to be 16 730 and 15 294 M^−1^ s^−1^ for *F. nucleatum* glutamate racemase for *S*- and *R*-Glu, while the corresponding values are 3000 and 3806 M^−1^ s^−1^ for the *B. subtilis* enzyme.^166^ In contrast, *M. tuberculosis* diaminopimelate epimerase^167^ has a very modest *k*_cat_/*K*_m_ of 883 M^−1^ s^−1^. On the other hand, RacX^168^ has extremely low *k*_cat_/*K*_m_ values of 2.86 and 3.23 M^−1^ s^−1^ for *S*- and *R*-Lys, while YgeA^168^ has *k*_cat_/*K*_m_ values of 45.8 and 45.8 M^−1^ s^−1^ for *S*- and *R*-His.

*k*_cat_/*K*_m_ values for other racemases and epimerases tend to be higher and this is often related to the lower *K*_m_ values observed for these larger substrates. For example, mandelate racemase (6.2 and 6.5 × 10^5^ M^−1^ s^−1^ for *S*- and *R*-mandelate^91^) and the *M. tuberculosis* homologue of AMACR (5.23 × 10^6^ and 6.0 × 10^6^ M^−1^ s^−1^ for *S*- and *R*-ibuprofenoyl-CoA,^146^ respectively). Finally, *N*-succinylamino acid racemases and *N*-acetylamino acid racemases exhibit highly variable *k*_cat_/*K*_m_ values (Table 1).

It is noteworthy that even the most efficient racemases/epimerases have *k*_cat_/*K*_m_ values well below the theoretical diffusion-controlled maximum of ∼1 × 10^9^ M^−1^ s^−1^.^169^ As proton-transfer reactions are extremely fast (between 5 × 10^9^ and 1 × 10^11^ M^−1^ s^−1^),^86^ rates may be limited by binding of substrate, release of product or conformational changes in the protein. A survey of *k*_cat_/*K*_m_ values for various enzymes^169^ shows that, for most enzymes, they are around 10^5^ to 10^9^ M^−1^ s^−1^, with the most efficient enzyme (superoxide dismutase) having a *k*_cat_/*K*_m_ of 7 × 10^9^ M^−1^ s^−1^. Moreover, *k*_cat_/*K*_m_ values for most enzyme-catalysed reactions appear to be diffusion-limited.^169^ There have been few detailed kinetic studies on racemases/epimerases but studies on mandelate racemase using mandelate as a substrate show that both *k*_cat_ and *k*_cat_/*K*_m_ are affected by increasing the viscosity of the solvent.^170,171^ This indicates that both binding of substrate and release of product are partly rate-limiting, although the effects on *k*_cat_ are more extensive than those on *k*_cat_/*K*_m_ indicating that that release of product is more sensitive to solvent viscosity than binding of substrate.^172^ In contrast, poorer substrates of mandelate racemase^91^ or less active mutants of the enzyme^173^ tend to be unaffected by increasing solvent viscosity, suggesting that rates are limited by the chemical reaction or other processes, *e.g.* conformational changes in the protein. Although the *k*_cat_/*K*_m_ values for mandelate racemisation is relatively modest (6.2 and 6.5 × 10^5^ M^−1^ s^−1^)^91^ compared to these other enzymes, it should be noted that racemisation of mandelate is a ‘difficult’ reaction as judged by the estimated half-life for the spontaneous uncatalysed reaction of 9.8 × 10^4^ year.^169^ Thus, mandelate racemase is providing a considerable enhancement (an effective molarity of ∼4.87 × 10^6^ M). It is unclear whether racemases/epimerases with lower *k*_cat_/*K*_m_ values are limited by diffusion, chemical reactivity or other processes, or whether these low efficiencies result from a low amount of active enzyme within the enzyme preparation.

## Drug design strategies for inhibiting racemases and epimerases

As noted above, many racemases and epimerases are drug targets for various diseases. The following is a survey of different strategies for the development of inhibitors.

### Substrate/product analogues

Exploiting the differences in side-chain conformation of different racemase/epimerase substrate stereochemical isomers can be a particularly fruitful strategy for the development of inhibitors. A significant advantage of these inhibitors is that they are achiral when identical sidechains are used. The substrate/product analogue approach works particularly well for racemases/epimerases possessing discrete side-chain-binding pockets for the different stereoisomers, *e.g.* mandelate racemase^139^ and *M. tuberculosis* α-methylacyl-CoA racemase (MCR).^149^ It can also work for enzymes with more subtle changes in side-chain conformation, *e.g.* aspartate racemase^174^ and glutamate racemase,^175^ although the potency of inhibition tends to be more modest. In many respects, substrate/product analogues are the equivalent of bisubstrate inhibitors of other enzymes,^176^ which often give rise to potent inhibition.

**Fig. 5:**
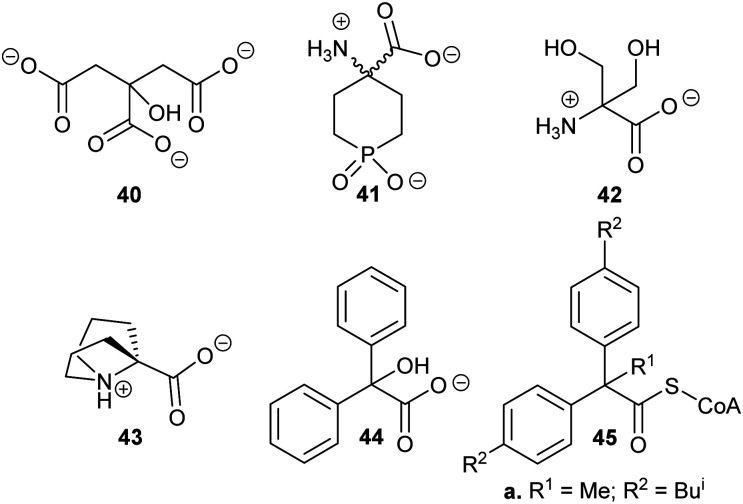
Structures of representative substrate–product analogues which are inhibitors of aspartate racemase (**40**),^174^ glutamate racemase (**41**),^175^ serine racemase (**42)**,^188^ proline racemase (**43**),^188^ mandelate racemase (**44**)^139^ and *M. tuberculosis* α-methylacyl-CoA racemase (MCR) (**45**).^149^

Several substrate/product analogues have been reported as inhibitors of amino-acid racemases (Fig. 5). For example, citrate **40** was shown by X-ray crystallography to bind as a substrate/product analogue to aspartate racemase.^174^ Citrate **40** behaves as a competitive inhibitor, although the potency was very low (*K*_i_ = 7.4 mM *vs. K*_m_ = 0.74 mM for l-aspartate). Pal *et al.* designed cyclic inhibitors of glutamate racemase, in which the ring mimicked the side-chain positions for the different stereoisomers of glutamate, including compound **41**.^175^ This proved to be a partial non-competitive inhibitor, although potency was modest (*K*_i_ = 3.1 mM *vs. K*_m_ = 1.41 mM for substrate *S*-glutamate).^175^ In contrast, substrate/product analogues were poor inhibitors of serine racemase (*e.g.***42**, mixed competitive inhibition; *K*_i_ = 167 mM and *K*_i_’ = 661 mM *vs. K*_m_ = 19 mM)^188^ and proline racemase (*e.g.***43**, non-competitive inhibition; *K*_i_ = 111 mM *vs. K*_m_ = 5.7 mM).^188^ Proline racemase is known to have an extremely confined active site in the ‘closed form’ of the enzyme,^26,27^ which binds substrates and inhibitors.

There have only been two substrate/product analogue studies on non-amino-acid racemases. Mandelate racemase substrate/product analogues^139^ bind with similar affinity to the substrate [*e.g.* benzilate (2,2-diphenyl-2-hydroxyacetate) **44**, *K*_i_ = 0.67 mM *vs. K*_m_ = 0.70 and 0.54 mM for *R*- and *S*-mandelate, respectively^139^]. Similarly, a substrate–product analogue of ibuprofenoyl-CoA (Fig. 5, **45a**) was a competitive inhibitor of the *M. tuberculosis* homologue of AMACR (MCR) and showed about a 6-fold increase in binding affinity (*K*_i_ = 16.9 μM *vs. K*_m_ = 106 μM) compared to ibuprofenoyl-CoA, undoubtedly due to the side-chain of the inhibitor binding to both the *R*- and *S*- subsites.^149^

### Enhancing acidity of the α-proton and alternative substrates

A number of racemases/epimerases have alternative substrates which undergo changes in stereochemical configuration^8,91,127,164,189^ or elimination.^63,82,120–124,128,161,164^ Efficiency of inhibition is dependent on concentrations of inhibitor and their catalytic efficiency as substrates (*k*_cat_/*K*_m_). Alternative substrates are usually competitive inhibitors (for example see^127^), which means that inhibition can be overcome by high concentrations of the substrate whose conversion is being inhibited.

**Fig. 6:**
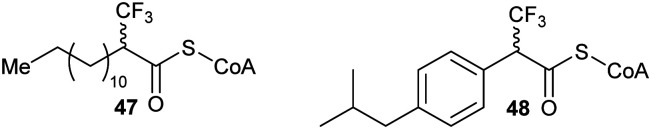
Representative inhibitors with increased C_α_–H acidity.^136,165,190^

Efficient inhibition can be achieved by increasing the acidity of the C_α_–H, *e.g.* by use of trifluoromethyl group (Fig. 6, **47** and **48**).^136,190^ The trifluoromethyl group lowers the energy of the enolate intermediate^81^ in the AMACR reaction;^136^ intermediates generally bind tightly to enzymes and more closely resemble the transition states of the reaction.^94^ The presence of a sulfur atom immediately adjacent to the substrate C_α_–H is also an effective strategy for increasing acidity (*vide infra*, Fig. 29).^165^

### Preventing the removal of the α-proton

**Fig. 7:**
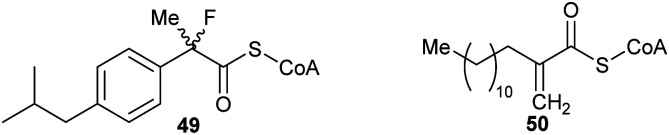
Representative inhibitors in which the C_α_–H is replaced.^191,192^

These types of inhibitors fall into two types: those in which the C_α_–H has been replaced by an alternative group and those with neighbouring groups which decrease the acidity of the C_α_–H. A number of different groups have been used to replace the C_α_–H (in addition to the substrate/product analogues with a second side-chain noted above), including fluorine atoms, *e.g.***49,**^191^ and methylene groups, *e.g.***50**^192^ (Fig. 7).

**Fig. 8:**
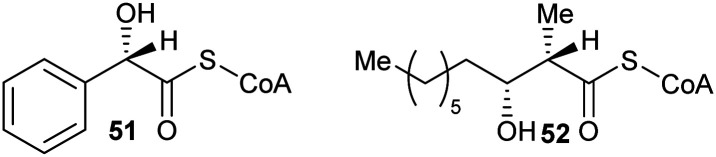
Representative inhibitors in which the acidity of C_α_–H is decreased.^164,191^

Inhibitors can also have substituents adjacent to the C_α_–H, which raise the energy of the deprotonated intermediate, such as hydroxy groups as exemplified by **51**^9,164^ and **52**^164^ (Fig. 8). Exchange of the C_α_–H was shown not to occur by incorporation studies in ^2^H_2_O and ^1^H NMR analyses when **51** and **52** were tested as substrates for AMACR.^9,164^ In all cases, these approaches tend to give rise to moderate inhibitors, as judged by the ratio of IC_50_/*K*_m_ or *K*_i_/*K*_m_ values.

### Transition-state and intermediate analogues

Transition-state analogues are widely recognised as potent drugs.^135,193,194^ This approach has been relatively under-used as a strategy for inhibition of racemases and epimerases, although the few examples show that highly potent inhibitors can be obtained.

**Fig. 9:**
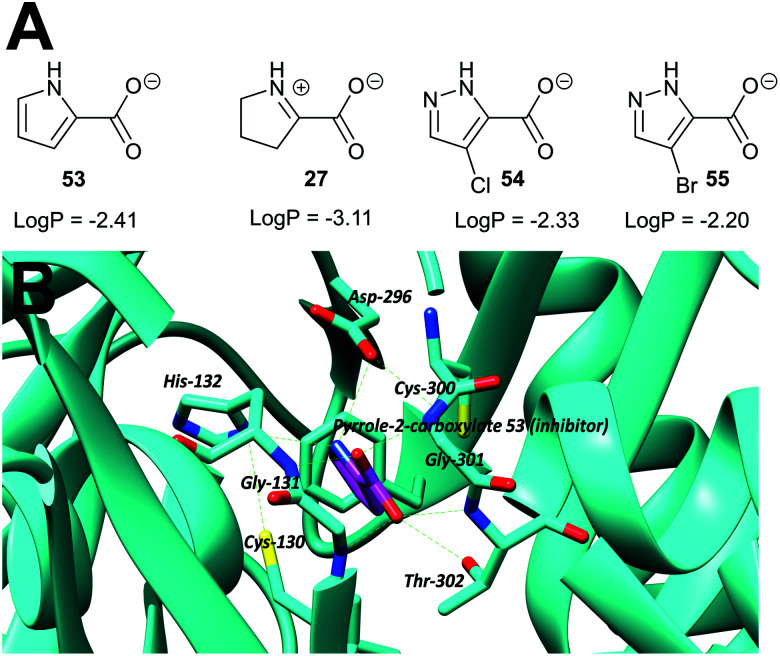
(A) Structures of proline racemase transition-state analogues.^135^ Log *P* values were calculated using: https://www.molinspiration.com/cgi-bin/properties. Log *P*, log_10_ (ratio of concentrations of drug in octan-1-ol and water at equilibrium); (B) X-ray crystal structure of pyrrole-2-carboxylate **53** bound within the active site of proline racemase from *T. cruzi*,^195^ showing the catalytic bases Cys-130 and Cys-300. Hydrogen bonds are shown as green dashed lines.

An early example is proline racemase, which is inhibited by pyrrole-2-carboxylate **53** and Δ-pyrroline-2-carboxylate **27** (reviewed in ref. 135) (Fig. 9A). Relatively high concentrations of these compounds are required for inhibition *in vitro* of the enzyme (about 10 × that of substrate) and they should be considered as inhibitors in which the C_α_–H is replaced (*vide supra*). X-ray crystallographic analysis showed that pyrrole-2-carboxylate binds within the *T. cruzi* active site (Fig. 9B) between the catalytic bases Cys-130 and Cys-300.^195^ However, despite its relatively low potency, pyrrole-2-carboxylate **53** reduced invasion of *T. cruzi* in infected mammalian cell models and also reduced differentiation of the parasite from the amastigote form into trypomastigotes.^28^ A number of more water-soluble analogues (*e.g.***54** and **55**) were tested for their ability to inhibit the enzyme but these proved to have lower potency.^26^ Compounds **53**, **54** and **55** had similar lipophilicity (calculated log *P* values of −2.41, −2.33 and −2.20, respectively) and the loss of inhibitory activity is likely to be related to the difficulties of accommodating the bulky halogen in the highly restricted active site. The halogen atom in **54** and **55** is also likely to force the carboxylate group out of plane, and this is expected to have a significant impact on binding affinity.

**Fig. 10:**
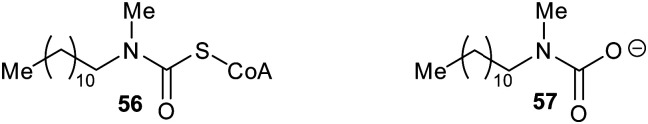
Structure of the enolate intermediate analogue as an inhibitor of AMACR^191^ and the unstable carbamate pro-drug.

A more recent example of the use of transition-state analogues are the carbamate inhibitors of α-methylacyl-CoA racemase (Fig. 10),^191^ which mimic the transition state (or enolate intermediate), giving rise to highly potent inhibition.^127,164,191^ Although the carbamate inhibitor **56** is by far the most potent AMACR inhibitor reported to date, it has limited utility because acyl-CoAs violate Lipinski guidelines and inhibitors are delivered to cells as the carboxylic acid pro-drug. Unfortunately, the acid pro-drug in this case would be a carbamate **57** which may readily decompose^164^ especially under acidic conditions or in the presence of cellular nucleophiles.

**Fig. 11:**
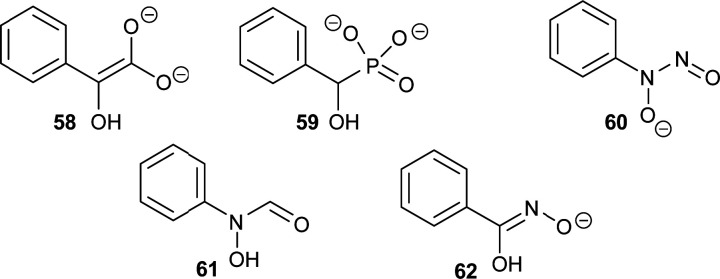
Inhibitors of mandelate racemase (**59–62**)^196–198^ resembling the aci-carboxylate deprotonated intermediate **58**.^196^

There are several other examples of using analogues of the deprotonated intermediate as inhibitors. For example, the conversion of mandelate by mandelate racemase is proposed to go through an aci-carboxylate intermediate (Fig. 11, **58**).^196^ Several mandelate racemase inhibitors of this type have been reported, including the phosphonate inhibitors^196,197^ such as the highly potent inhibitor **59** (*K*_i_ = 4.7 μM *vs. K*_m_ of 1.0 and 1.2 mM for *R*- and *S*-mandelate, respectively). The phosphonate group in **59** possesses two negatively charged oxygen atoms, and hence resembles the aci-carboxylate intermediate **58**. Similarly, cupferron **60** and *N*-hydroxyformanilide **61** also act as analogues of the deprotonated intermediate **58** because they have an extended planar system of sp^2^-hybridised atoms, whilst benzohydroxamate **62** is a hydroxamate. Inhibitors **59–62** (Fig. 11) strongly ligate to the metal in the active site of mandelate racemase (*K*_i_ values of 2.7, 2.8 and 9.3 μM, respectively).^198^

### Allosteric inhibition

Allosteric inhibition arises from inhibitors binding somewhere other than at the enzyme active site. The uncompetitive type of inhibition observed through enzyme kinetics arises from binding of the inhibitor to the enzyme-substrate complex with (almost) no binding to unoccupied enzyme^199^ and, hence, must arise from binding at an allosteric site.

**Fig. 12:**
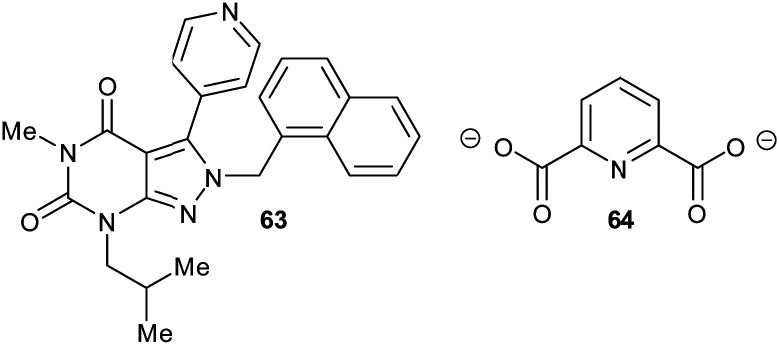
Structures of the allosteric inhibitors **63** (*H. pylori* glutamate racemase)^200^ and pyridine-2,6-dicarboxylate **64** (*B. anthracis* glutamate racemase).^25^ The ionisation state of **64** which is shown is that used in the virtual screen.

Glutamate racemase is the only racemase/epimerase for which confirmed allosteric inhibitors have been reported. Lundqvist *et al.* identified an uncompetitive inhibitor **63** during a high-throughput screening campaign on the *H. pylori* enzyme (Fig. 12).^200^ The inhibitor-binding site is remote from the active site.^21,200^ A second cryptic inhibitor-binding site was subsequently identified in the *B. anthracis* enzyme by virtual screening, which led to the identification of pyridine-2,6-dicarboxylate (dipicolinate) **64** as an inhibitor (Fig. 12), with *K*_i_ = 1.9 mM.^25^ Further studies on **37** showed that inhibitor binding resulted in the active-site Cys^185^ adopting a conformation in which the SH group points away from glutamate C_α_–H.^21^ It is also noted that some uncompetitive inhibitors of α-methylacyl-CoA racemase were recently identified (*vide infra*, Fig. 15),^163^ implying that they bind to an allosteric site, although the exact binding site has not been confirmed.

### Covalent inhibition

Inhibitors which form a covalent bond to their targets are enjoying a resurgence because of their potential for long-lasting effects and strong affinity for the target, amongst other benefits.^201–207^ Indeed, around 30% of all approved clinical drugs acting on enzymes are covalent inhibitors.^202,207^ Covalent inhibitors can cause either reversible or irreversible inhibition of their target.^204–208^ There is a perception that covalent inhibitors are non-selective and hence are less useful. However, studies have shown that high selectivity for the target enzyme can be achieved.^203–210^ Modification of the substituents around the electrophile can also further enhance selectivity,^210–212^ especially for electrophiles modifying cysteine residues^212–215^ (which are the catalytic bases in many cofactor-independent amino-acid racemases and epimerases^5,18,20,22^). Electrophilic properties can be predicted using the ‘electrophilicity index’.^209^

**Scheme 10:**
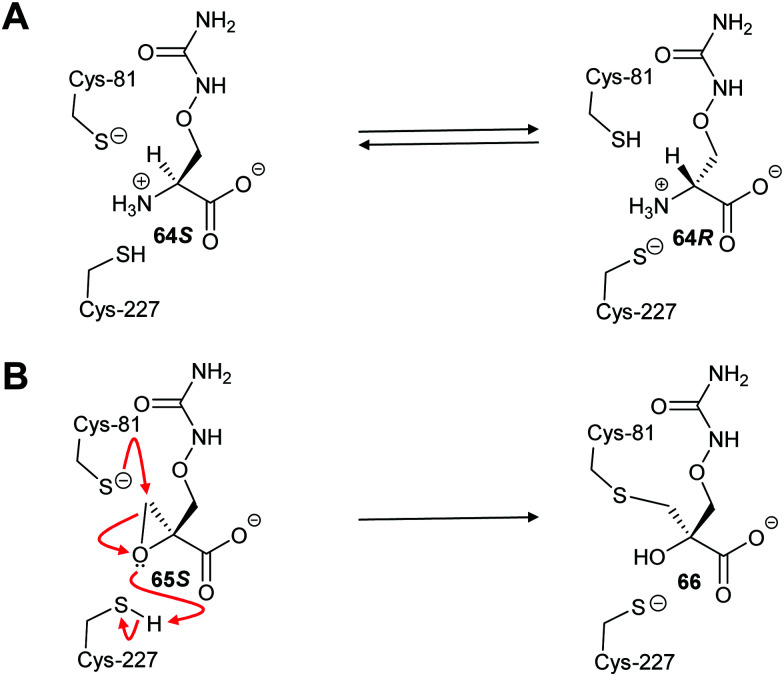
(A) Interconversion of *O*-ureidoserine substrate isomers **64S** and **64R** by *O*-ureidoserine racemase. (B) Inactivation of *O*-ureidoserine racemase by epoxide **65S** to give covalent adduct **66.** The roles of the Cys residues are reversed for the enantiomeric epoxide **65R**.^46^

Covalent inhibition of racemases and epimerases has been previously investigated. Both diaminopimelate epimerase^20,124^ and α-methylacyl-CoA racemase^127,152^ have been shown to be inhibited by non-specific protein-modification agents. In each case, these are cysteine-reactive compounds such as iodoacetamide, ebselen (2-phenyl-1,2-benzoisoselenazol-3(2*H*)-one) and ebselen oxide. It is also noted that mandelate racemase undergoes covalent inhibition by 3-hydroxypyruvate because of formation of an imine between the inhibitor and Lys-166, one of the active-site bases.^216^ There have also been several attempts to design irreversible inhibitors rationally, most notably the aziridine inhibitors of diaminopimelate epimerase.^20,124,217,218^ A recent example of rational covalent inhibitor design is seen with *O*-ureidoserine racemase (which interconverts *S*- and *R*- *O*-ureidoserine **65**), which is irreversibly inhibited by oxiranes *R*- and *S*-**66** to give covalent adducts (Scheme 10).^46^

**Scheme 11:**
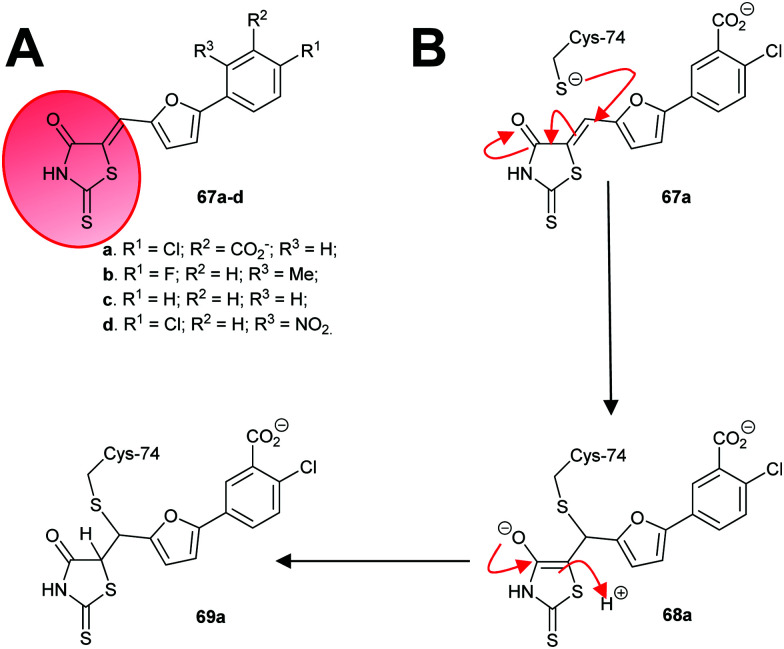
(A) Structures of irreversible inhibitors of *B. subtilis* glutamate racemase.^22^ The rhodanine motif is highlighted in red; (B) inactivation of glutamate racemase by **68a** by 1,4-conjugate addition.

An irreversible inhibitor of *B. subtilis* glutamate racemase was also identified by virtual screening (*vide infra*),^23,24^ and was proposed to bind close to the catalytic cysteine residues.^22^ The inhibitor is proposed to modify irreversibly one of these thiols by conjugate addition (a.k.a. Michael addition).^202,205,206^ The hit compound **67a** and several analogues **67b–67d** (Scheme 11A) were subsequently shown to be irreversible inhibitors.^22^ Compounds **67a** and **67c** proved to be non-saturating inhibitors. In contrast, **67b** and **67d** displayed saturating inhibition, consistent with modification of active site residues. Further experiments showed that inhibition was reversible, consistent with a reversible conjugate addition *via* enolate **68a** to give the product **69a** (Scheme 11B). Mass spectrometric analysis of wild-type and C74A mutant glutamate racemase following incubation with **67a** confirmed modification of Cys-74, one of the active-site bases. Compound **67a** was unreactive with 2-mercaptoethanol under the assay conditions,^22^ showing that conjugate addition to thiols only occurred in the presence of the high nucleophilic Cys-74 in the enzyme active site. The rhodanine warhead (Scheme 11A) is recognised as a common motif found in pan-assay interference compounds (PAINs), which give rise to false positive or intractable leads in high-throughput screening campaigns.^219^ These rhodanine glutamate racemase inhibitors showed activity against various bacterial strains, including various methicillin-resistant *S. aureus* strains.^22^

In a second study, 3-chloroalanine **70** (β-chloroalanine; a poor inhibitor of PLP-dependent alanine racemase^134^) was shown to irreversibly inactivate glutamate racemase from *M. tuberculosis*.^134^ Non-saturating kinetics where observed for the *S*-isomer with a second-order rate constant of 2.7 M^−1^ s^−1^. Mass spectrometric analysis of peptides showed that 3-chloro-*S*-alanine (**70S**) reacted at Cys-185, while 3-chloro-*R*-alanine (**70R**) reacted at Cys-74. In the glutamate racemase reaction, *R*-glutamate is deprotonated by Cys-74 whilst *S*-glutamate is deprotonated by Cys-185 during enantiomerisation, *i.e.* the active-site Cys acting as an acid is derivatised by 3-chloro-alanine **70**.

**Scheme 12:**
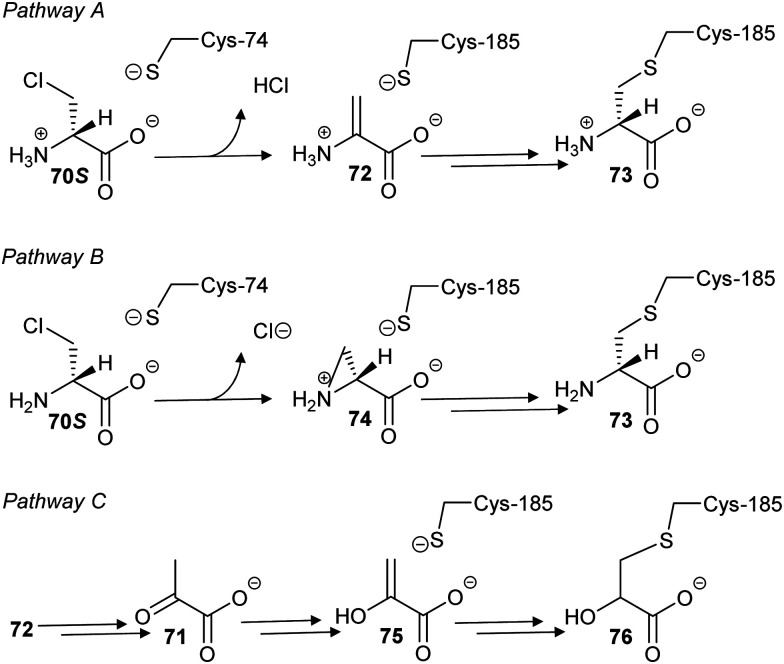
Proposed mechanisms of glutamate racemase inactivation by 3-chloro-*S*-alanine **70S** (β-chloro-*S*-alanine).

The authors proposed^134^ that the adduct was a pyruvate derivative, based on the observation that pyruvate **71** was generated upon treatment of the enzyme with 3-chloro-alanine **70** but their proposed mechanism is very unlikely. Two more likely mechanisms can be envisaged (Scheme 12, pathways A and B) based on the observed increase in mass of ∼87 Da. In pathway A, removal of the C_α_–H of **70S** by Cys-74 results in elimination of HCl, yielding the aminoacrylate complex **72**. This is followed by conjugate addition of Cys-185 to give **73**. However, complex **72** is achiral and non-specific derivatisation of the active site Cys residues might be expected if **72** resulted in alkylation. Pathway B, *via* the aziridine intermediate **74**, preserves the chirality of reaction and gives rise to the same adduct **73**. However, the active site Cys residues are relatively distant from the α-amino group, making pathway B less likely. Digestion of the derivatised enzyme and mass spectrometric analysis shows the presence of nitrogen within the enzyme-inhibitor adduct, discounting the possibility that the adduct is a pyruvate derivative (Scheme 12, pathway C). The observed pyruvate **17** generated in the reaction arises from tautomerisation of aminoacrylate **72** to the imine followed by hydrolysis, *i.e.***70** behaves as a substrate as well as an inhibitor.^220^

**Scheme 13:**
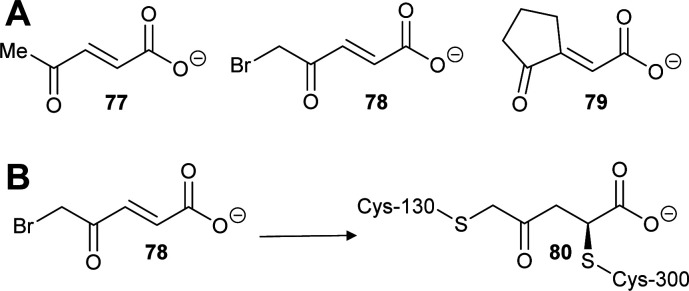
(A) Structures of highly active covalent inhibitors of proline racemase;^26,27^ (B) Reaction of **78** with catalytic cysteine residues in proline racemase by conjugate addition and S_N_2 reaction to give a cross-linked adduct **80**.^27^

Similarly, two covalent inhibitors (**77** and **78**) of *T. cruzi* proline racemase were identified by virtual screening.^26^ The inhibitors were proposed to modify the active-site cysteine residues^26^ by conjugate addition^202,205,206^ and this was subsequently confirmed by X-ray crystallography.^27^ The active compounds (Scheme 13A) each have a double bond in conjugation with a carboxylate and a ketone^26,27^ and X-ray crystallography showed that conjugate addition occurred towards the ketone.^27^ This is unsurprising as ketone carbons are more δ+ than carboxylic acids/carboxylates and hence conjugate addition is expected to occur towards the ketone. The most active compound of those subsequently investigated (NG-P27, **79**)^27^ was active against *T. cruzi* in infected mammalian cells. It is also notable that one of the original compounds^26^ (5-bromo-4-oxopent-2-enoate **78**) is divalent and reacts with both active-site cysteine residues, cross-linking the enzyme to give adduct **80** (Scheme 13B).^27^

### Virtual screening and structure-based fragment screening

Virtual screening of drug targets with a compound library is a well-established method in drug discovery.^221–225^ These approaches can utilise artificial intelligence to optimise the process^223^ or negative design^222^ to remove compounds which are poor prospects.

**Fig. 13:**
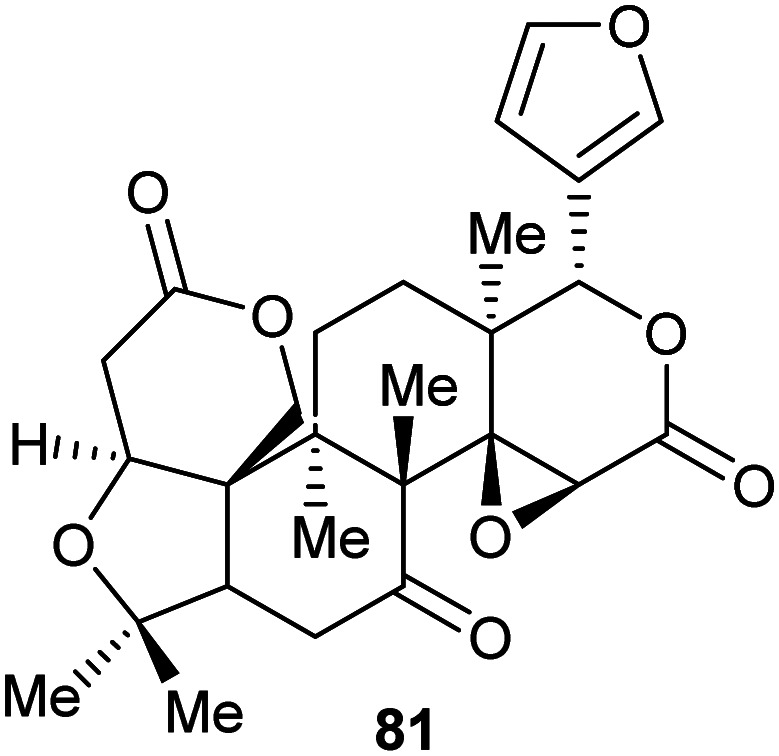
Structure of limonin **81**.^226^

There are only a few examples of virtual screening being used for identification of inhibitors of racemases/epimerases and all have been for amino-acid racemases. For example, Skariyachan *et al.* conducted a screen of a virtual natural products library against diaminopimelate epimerase, amongst several other targets, identifying limonin **81** as a hit (Fig. 13).^226^ Limonin **81** and several other hits showed dose-dependent activity against a clinical strain of multi-drug resistant *Acinetobacter baumannii*.

**Fig. 14:**
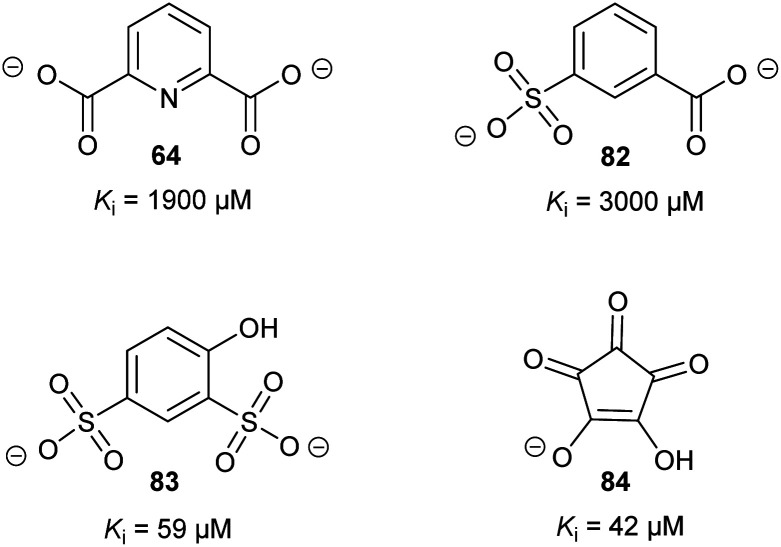
Representative inhibitors of *B. subtilis* glutamate racemase identified using virtual high-throughput screening.^23,24^ The inhibitor ionisation state shown are those that were used in the virtual screen.

Studies on *B. subtilis* glutamate racemase used *ab initio* quantum mechanics/molecular mechanics to probe transition states in the reaction.^23^ A strong correlation between computational and experimentally determined binding of known inhibitors was observed. The same study^23^ used a enzymatic transition state conformation in a virtual screen of over one million compounds followed by experimental testing. Although no tight-binding inhibitors were identified, several common motifs for competitive inhibitors were identified. A subsequent virtual screening study on the same enzyme^24^ identified several competitive inhibitors such as **64** and **82**, with the two most potent inhibitors, **83** and **84**, having *K*_i_ values of 59 and 42 μM (Fig. 14).

**Fig. 15:**
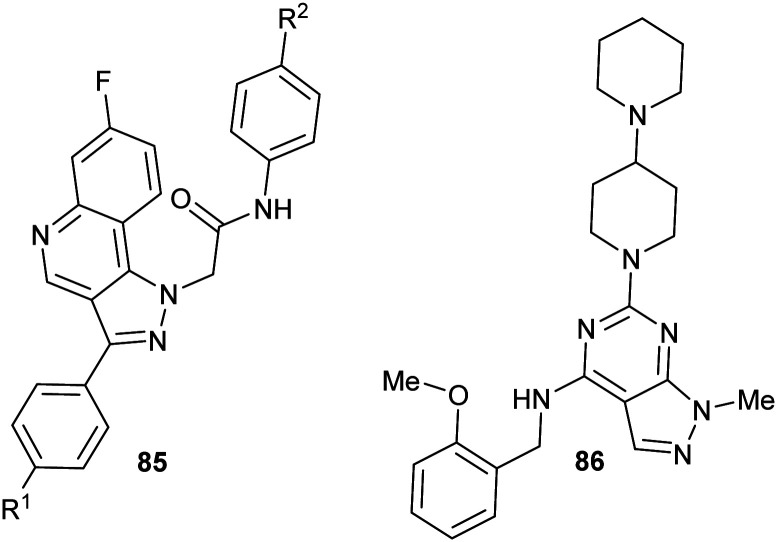
Representative structures of pyrazoloquinoline and pyrazolopyrimidine AMACR inhibitors.^163^**85a**, R^1^ = R^2^ = H, uncompetitive inhibition, *K*_i_ = 4.8 ± 0.7 μM; **85b**, R^1^ = R^2^ = F, mixed competitive inhibition, *K*_i_ = 2.4 ± 0.9 μM; **86**, uncompetitive inhibition, *K*_i_ = 4.6 ± 0.4 μM.

Virtual screening has also been used against proline racemase from *T. cruzi*, the causative agent of Chagas disease (*vide supra*).^26^ Proline racemase without a ligand exists in an ‘open’ conformation. Upon binding of an inhibitor, a ‘closed’ conformation is adopted, such that a very restricted active site is produced, which prevents design of inhibitors using standard approaches. The virtual screening study generated forty-nine intermediate conformations *en route* from the ‘open’ to ‘closed’ conformations. Four of these conformations were used in virtual screens of 31 000 compounds. These screens led to the identification of covalent inhibitors (*vide supra*, Scheme 13), which showed dose-dependent activity against *T. cruzi* in infected mammalian cells.^26,27^

### High-throughput screening and related approaches

High-throughput screening is an under-utilised approach to discovering inhibitors of racemases and epimerases. High-throughput screening offers a number of advantages, including the possibility of discovering inhibitors which are not competitive (which is the mode of inhibition often observed for active-site-directed inhibitors).^227^

Several different *in vitro* assays have been used in discovery campaigns. Release of tritium (^3^H^+^) from a radiolabelled substrate into solvent was used in a screen of ∼5000 compounds against human α-methylacyl-CoA racemase (AMACR).^152^ Crucially, the assay requires several steps, including chromatographic separation of residual (acyl-CoA) substrate from tritiated water product. Therefore, this assay is not ideally suited for high-throughput or fragment-screening campaigns. This study^152^ identified a number of non-specific protein-modifying and degrading agents, such as ebselen, ebselen oxide and Rose Bengal.^127,152^

A subsequent high-throughput screen on human AMACR^163^ made use of an eliminating substrate **37** (*vide supra*, Scheme 9) producing 2,4-dinitrophenoxide **38**.^127^ Conveniently, this allowed identification of inhibitors based on absorbance changes over the time course in a continuous assay. The screen identified a series of mixed competitive and uncompetitive pyrazoloquinolines, *e.g.***85**, and pyrazolopyrimidines, *e.g.***86** (Fig. 15). The use of a chromogenic substrate^127^ allows real-time monitoring of the enzymatic reaction but substrates of this type can only be used with a few racemases/epimerases.

Two studies have made use of high-throughput screens with coupling enzymes. The first study, by Lundqvist *et al.*, conducted a high-throughput screen of 385 861 compounds against *H. pylori* glutamate racemase.^200^ No details of the actual screen are given but the authors used two different assays for assessing identified inhibitor activity: conversion of *S*- to *R*-glutamate was coupled to UDP-*N*-acetyl-muramic acid-alanine: *R*-glutamate ligase (MurD) and purine nucleoside phosphorylase with monitoring of the reaction at 360 nm. In the second assay, conversion of *R*- to *S*-glutamate was coupled to *S*-glutamate dehydrogenase with spectrophotometric monitoring of conversion of NAD^+^ to NADH. These screens led to the identification of an uncompetitive inhibitor (*vide supra*Fig. 12, **63**), which was subsequently shown to exert its effect by changing the conformation of the catalytic base, Cys-185, such that it points away from the glutamate substrate C_α_–H.

**Fig. 16:**
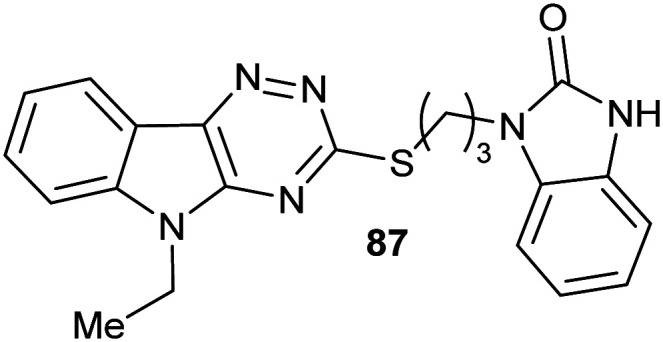
Structure of the most potent hit **87** identified by high-throughput screening inhibiting RmlC. **61** is a fully reversible, competitive inhibitor with IC_50_ = 200 nM.^228^

In a second example^228^ of a coupled assay, dTDP-6-deoxy-d-xylo-4-hexopyranosid-4-ulose 3,5-epimerase (RmlC) was coupled to the subsequent enzyme in the biosynthetic pathway, which is NADP^+^-dependent, with activity being followed by decreasing fluorescence of NADPH at 460 nm. This led to the identification of a series of inhibitors, including some with potency in the nM range *e.g.***87** (Fig. 16).^228^ Use of coupling enzymes, such as in these examples, is a standard approach in high-throughput and fragment-screening campaigns^227^ (*vide infra*), although it is always necessary to check if the hits are inhibiting the desired target or the coupling enzyme.

Racemases used in biotechnological applications have been assayed in several different ways, typically using oxidase enzymes of various types. These assays could be adapted for high-throughput screening for inhibitors. For example, mutant mandelate racemases were assayed using mandelate dehydrogenase, which uses NAD^+^. Conveniently, the ketoacid product can be assayed using 2,4-dinitrophenylhydrazine (2,4-DNPH) at alkaline pH with the final 2,4-dinitrophenylhydrazone product absorbing at 450 nm.^54^ Ketoacids are also be produced by the action of amino-acid racemases and epimerases on amino-acid hydroxamate and other eliminating substrates^82,128^ (*vide supra*, Schemes 3, 4, 6 and 7). Alternatively, the NAD(P)H product from dehydrogenases can be assayed using diaphorases^23,125^ or by direct monitoring of absorbance or fluorescence.

**Scheme 14:**
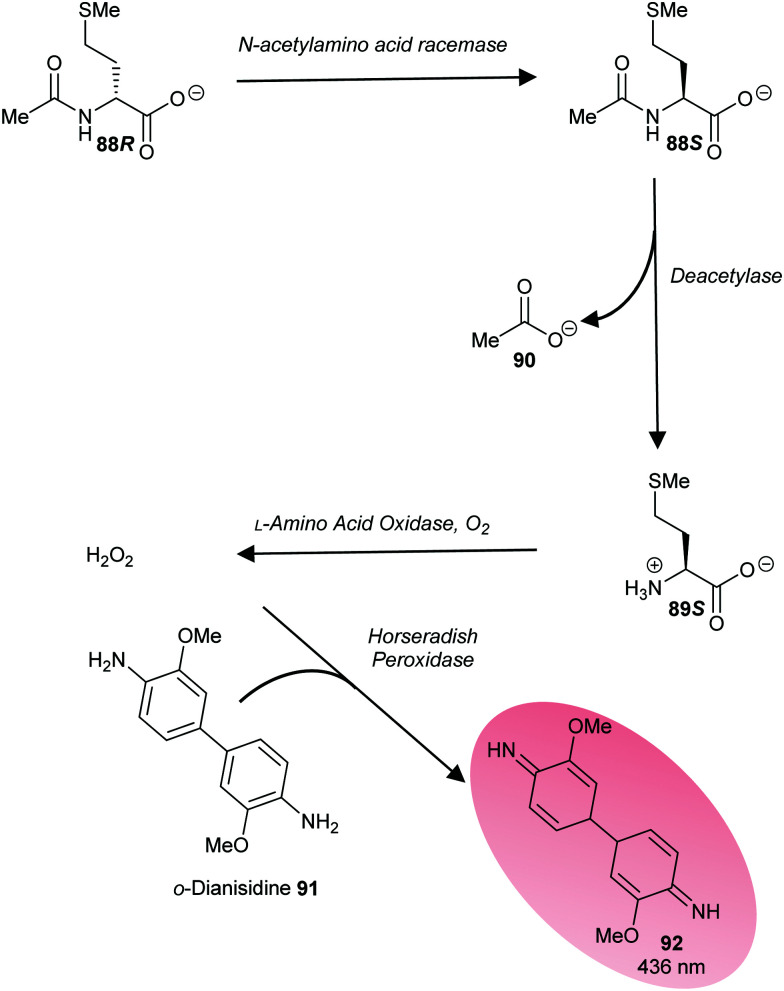
Continuous assay of *N*-acetyl-amino acid racemases using a three-enzyme coupling system to give a coloured product **92** absorbing at 436 nm.^181^

Similarly, hydrogen peroxide is produced by several oxidative enzymes, including d-amino-acid oxidase, which can be conveniently assayed using horseradish peroxidase.^174,229^ Notably, an assay based on d-amino-acid oxidase/horseradish peroxidase was used to evaluate rationally designed inhibitors of proline racemase^26^ and in the high-throughput screening of alanine racemase.^230^ Similarly, a continuous assay for *N*-acetylamino-acid racemases was developed using the *R*- substrate **88R** (Scheme 14).^181^ A stereoselective deacetylase was used to convert the *S*- product **88S** to the corresponding *S*-amino acid **89S** and acetate **90**. l-Amino acid oxidase was used to produce H_2_O_2_ from **89S**, which was quantified by the horseradish peroxidase-catalysed oxidation of dianisidine **90** to give the coloured oxidation product **91**.

In additional, several racemases/epimerases eliminate HF from fluorine-containing substrates (*vide supra*, Scheme 8),^120,121^ which could potentially be assayed using fluoride sensors, although this can be challenging in aqueous systems.^161^ Finally, microscale, medium-throughput polarimetric assays offer the possibility of direct observation of the change in chirality^142^ during screening.

## Inhibitors of racemases and epimerases

Racemases and epimerases play pivotal roles in metabolism and are excellent drug targets. The following is a survey of recent advances in drug development, focussing on recently reported small-molecule inhibitors. Inhibition of many amino-acid racemases/epimerases has been the subject of a recent excellent review^20^ and readers are referred to this and the above sections for details of studies on inhibition of diaminopimelate racemase,^20,124,217,218,226^ proline racemase,^26,27,135,188^ hydroxyproline epimerase,^20^ aspartate racemase,^20^ serine racemase,^188^ isoleucine epimerase^20^ and *O*-ureidoserine racemase.^46^ Studies on inhibition of mandelate racemase^91,139,196–198,216^ are detailed in the section on inhibition strategies above. There have been no reported studies on inhibition of *E*cL-DER, RacX, YgeA, McyF, YcjG or *N*-acetylmannoseamine-6-phosphate 2-epimerase or *N*-acetylamino-acid or *N*-succinylamino-acid racemases since 2015.

### PLP-dependent racemases

#### Alanine racemase

Alanine racemase is involved in cyclosporine biosynthesis^179^ and is a well-established antibacterial drug target.^18,20^ The enzyme has been the subject of extensive inhibitor studies,^231,232^ including the early studies with 3-fluoroalanine and 3-chloroalanine noted above.^132^ Other inhibitors include several peptide and halogen-containing peptides, phosphonic acid derivatives (fosfalin) and various halovinylglycines and thiadiazolidinones.^231,232^

**Scheme 15:**
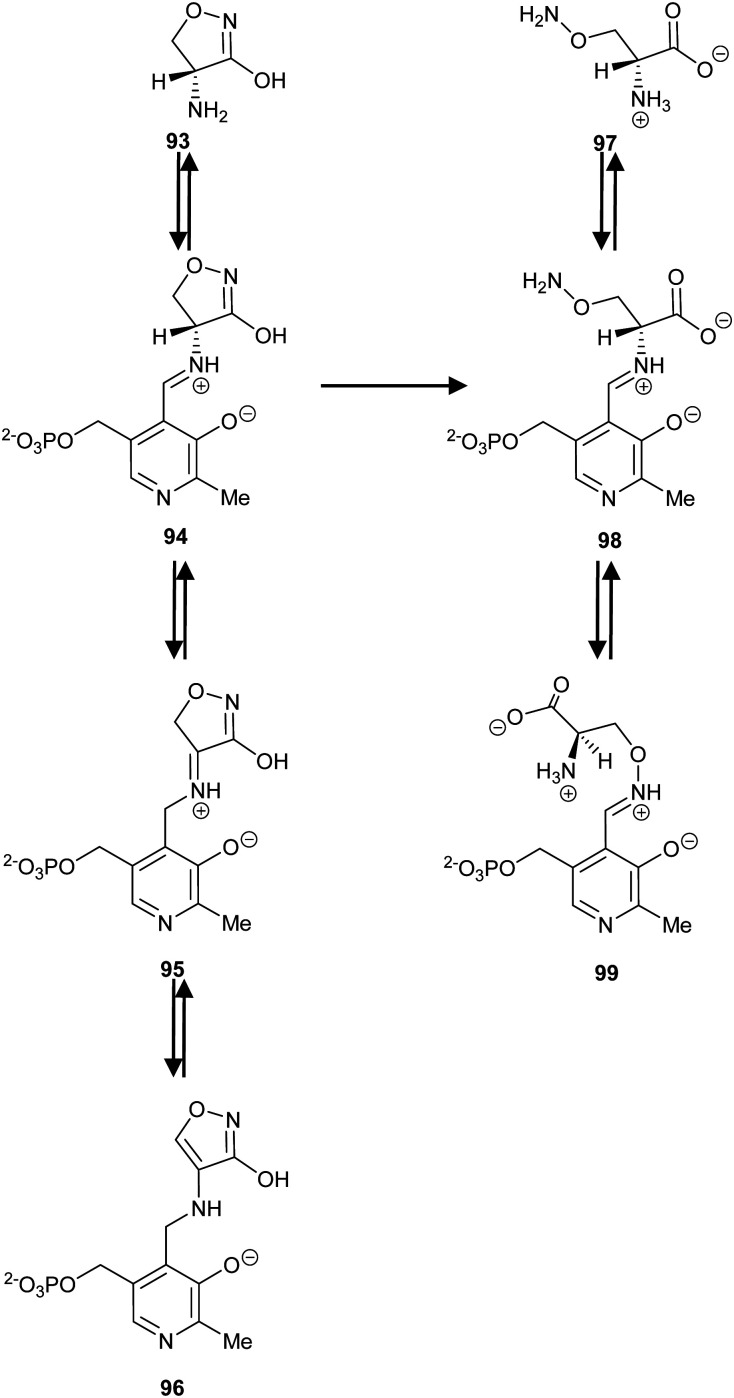
Inhibition of alanine racemase by d-cycloserine **93**. Note that **96** was proposed to be directly converted into **99**^233^ but it is more likely that **94** undergoes hydrolysis to form **98**, which releases **97**. This directly forms two different external aldimines, **98** or **99**, respectively (see text for details).

An important inhibitor of alanine racemase is d-cycloserine, a natural product used in the treatment of drug resistant tuberculosis.^233,234^ It is notable that the biosynthetic pathway for d-cycloserine contains a reaction catalysed by a racemase, *O*-ureidoserine racemase^46^ (*vide supra*, Scheme 10A). d-Cycloserine **93** is a relatively non-specific antibiotic and targets *M. tuberculosis* alanine racemase and d-Ala-d-Ala ligase.^233,234^ Inhibition of alanine racemase is proposed to occur by formation of the external aldimine **94** followed by reversible rearrangement to the ketimine **95** and isoxazole **96** (Scheme 15).^233^ Stereoselective isotope exchange with solvent is observed when the reaction is carried out in ^2^H_2_O, with d-cycloserine **93** incorporating deuterium at the α-position without a change in stereochemical configuration. Deuteration appears to arise by exchange of the α-proton. Incubations of alanine racemase with d-cycloserine **93** also result in the formation of isoxazole **97**.^233^ The authors propose a complex rearrangement of the keto tautomer of **96** but this seems unlikely. A simpler explanation is that alanine racemase catalyses hydrolysis of aldimine **94** using a hydrogen-bonded water molecule, to form the linear aldimine **98** directly. This could undergo imine exchange to form **99** but a more likely scenario is that **97** is released from **98** which uses its hydroxylamine to form the aldimine complex **99** directly (the p*K*_a_ for the conjugate acid of the α-NH_2_ and γ-O-NH_2_ groups are 9.14 and 3.16,^235^ respectively, and, hence, the γ-O-NH_2_ group will be uncharged at neutral pH). Thus, d-cycloserine **93** is both a substrate (undergoing α-proton exchange and hydrolysis) and an irreversible inhibitor of alanine racemase, consistent with the observation that *M. tuberculosis* alanine racemase is not fully inhibited even by high concentrations of **93**.

**Fig. 17:**
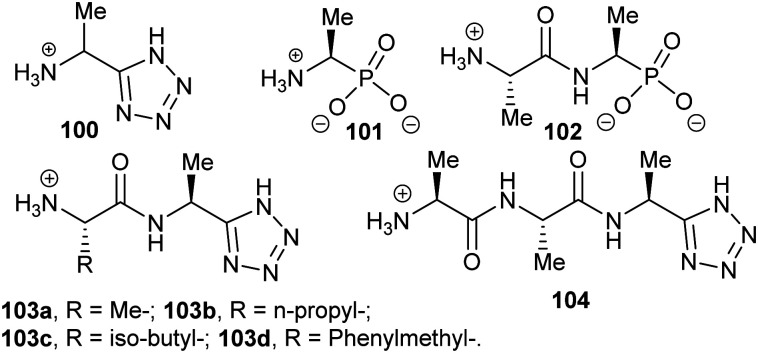
Structures of 5-(1-aminoethyl)tetrazole **100**, fosfalin **101**, alafosfalin **102**, and dipeptide analogues **103a-d** and tripeptide analogue **104**.^236^

A series of tetrazole-peptide derivatives were also designed and synthesised as inhibitors of alanine racemase in bacterial cells (Fig. 17).^236^ The tetrazole group is a well-established bioisostere of the carboxylate group,^236^ and hence 5-(1-aminoethyl)tetrazole **100** should behave as an analogue of alanine. *S*- and *R*-5-(1-aminoethyl)tetrazole **100** (AET) were inactive when tested against a series of Gram-negative and -positive bacteria^236^ but this is unsurprising as it is known that alanine is imported into bacterial cells as an oligopeptide. Indeed, fosfalin **101** is delivered to bacterial cells as a “dipeptide” alafosfalin **102**.^231,236^

Therefore, a series of di- and tripeptide derivatives were synthesised and tested.^236^ The *SS*-Ala-Ala analogue **103a** was active against several Gram-negative species, whilst the *SS*-norvalene-AET **103b**, *SS*-Leu-AET **103c** and *SS*-Phe-AET **103d** analogues were active against several Gram-positive species. The *SSS*-Ala-Ala-AET analogue **104** showed similar activity to **103a**. *N*-Succinyl derivatives of these peptide analogues were largely inactive. It was not determined if *S*- and *R*-5-(1-aminoethyl)tetrazole **100** or any of the peptide analogues were inhibitors of alanine racemase and hence the mechanism of antibacterial activity has not been confirmed.

**Fig. 18:**
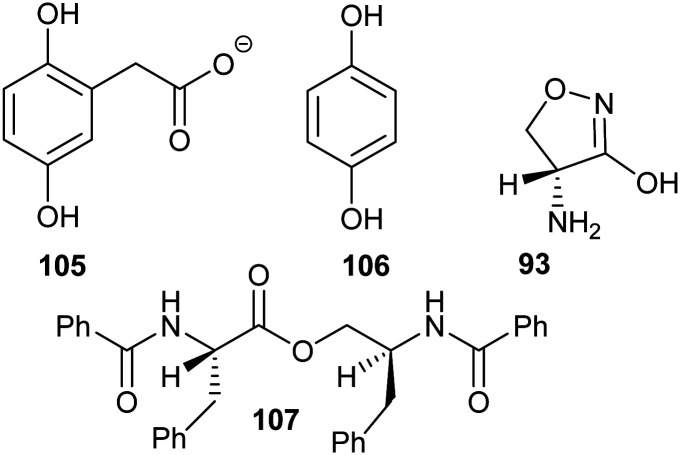
Structures of alanine racemase inhibitors identified by high-throughput screening.^230^ Abbreviation used: Ph, phenyl.

High-throughput screening of small-molecule and fungal extract libraries against *Aeromonas hydrophila* alanine racemase has also been performed. This screen identified several previously unknown inhibitors (Fig. 18) of moderate potency (IC_50_ = 6.6 to 18.5 μM), including homogentisic acid **105** and hydroxyquinone **106**.^230^d-Cycloserine **93** (the control inhibitor) had an IC_50_ of 5.4 μM under the conditions used in this screen. Kinetic analysis showed that homogentisic acid **105** was a competitive inhibitor (*K*_i_ = 51.7 μM) whilst hydroxyquinone **106** was a non-competitive inhibitor (*K*_i_ = 212 μM). These two compounds showed antibacterial activity against *A. hydrophila*, a Gram-negative anaerobic pathogen. Anabellamide **107** was a potent inhibitor of alanine racemase *in vitro* (IC_50_ = 6.6 μM) but was inactive in cellular assays (Fig. 18).

#### Isoleucine 2-epimerase

Isoleucine 2-epimerase is a novel anti-bacterial drug target. There are only two studies on the inhibition of isoleucine 2-epimerase.^180,189^ Mutaguchi *et al.* noted, in their original characterisation of the enzyme, that it was inhibited by non-specific inhibitors of other PLP-dependent enzymes, such as hydroxylamine, aminooxyacetate and phenylhydrazine.^180^ The effect of hydroxylamine was reversed upon dialysis and addition of PLP to the buffer, providing good evidence that the enzyme is a PLP-dependent epimerase.

**Fig. 19:**
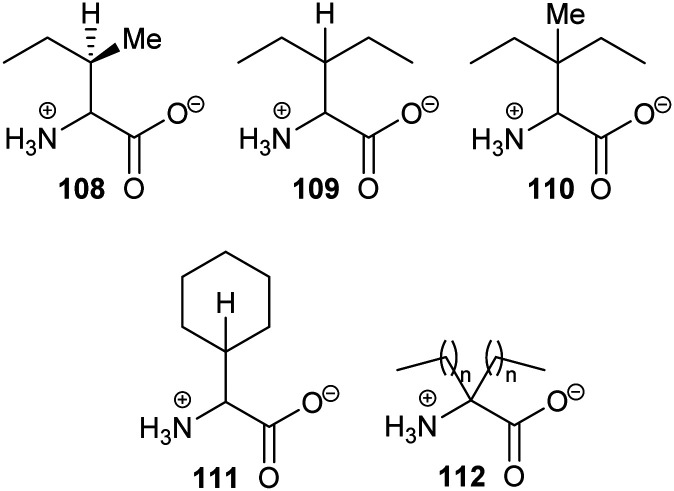
structures of isoleucine **108** and inhibitors of isoleucine 2-epimerase.^189^ For **112**, *n* = 1, 2 and 3.

Subsequently, a study investigating inhibition of *Lactobacillus buchneri* isoleucine 2-epimerase by substrate/product analogues was reported.^189^ Two groups of inhibitors (Fig. 19) were investigated, based on the structure of the substrate *S*-isoleucine **108**. These inhibitors fell into two classes: those with a single modified sidechain (**109–111**); and those with dual sidechains (**112**), which are similar to the substrate–product analogues that inhibit other racemases and epimerases.^139,149,150,174,188,197,198^ The 2*R*- and 2*S*- enantiomers of **109** were substrates of the enzyme, although these are converted with an efficiency of only ∼50% to 80% of that of the natural substrates (as judged by *k*_cat_/*K*_m_ values^180,189^) and, hence, would not be very effective competitive inhibitors. On the other hand, **110** which possesses an additional methyl group on the sidechain was not a substrate but instead behaved as a pure competitive inhibitor (*K*_i_ values of 1.5 and 2.9 mM for the 2*R* and 2*S* enantiomers, respectively). Compound **111** which has a cyclic sidechain was also an alternative substrate and was converted with very similar efficiencies to **109** (as judged by *k*_cat_/*K*_m_ values). The synthesised compounds with dual sidechains (**112**, *n* = 1, 2 and 3) were rather poor inhibitors, although potency increased with increasing size and hydrophobicity of the sidechain (*K*_i_ = 144, 19 and 11 mM, respectively).

#### Serine racemase

Serine racemase catalyses the formation of *R*-serine from *S*-serine as well as the elimination of water.^69^*R*-Serine binds to the *N*-methyl-d-aspartate (NMDA) receptor glycine-binding site. The NMDA receptor is associated with several neurological diseases, including Alzheimer's disease, amyotrophic lateral sclerosis, Huntington's disease, Parkinson's disease, epilepsy and eye disease, amongst others, and psychiatric diseases such as schizophrenia, and depression.^35,237–241^ Hence, inhibition of serine racemase is of interest as a strategy for the manipulation of levels of *R*-serine.

**Fig. 20:**
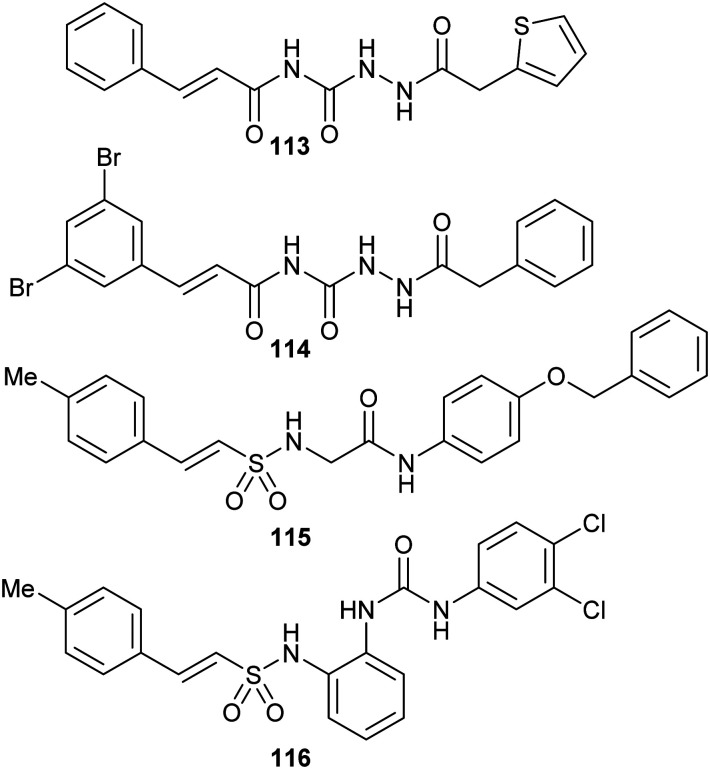
Structures of inhibitors of serine racemase.^239,245^

A large number of studies have appeared on the biological and pathological role of serine racemase in the last five years^35,69,129,241–244^ but only one study has reported synthesis and evaluation of inhibitors.^239^ This study^239^ elaborated a potent hit (Fig. 20, **113**) identified in a previous virtual screen.^245^ Of the synthesised compounds, five showed potent inhibition of serine racemase *in vitro*. Two of these compounds were similar in structure to the original hit and IC_50_ values were determined for the three other compounds (140, 270 and 280 μM for **114**, **115** and **116**, respectively). This compares to an IC_50_ value of 770 μM for malonate, a standard inhibitor. Further studies showed **114** reduced NDMA receptor activation by ∼1.4-fold, consistent with engagement of the target *in vivo*.

### Cofactor-independent racemases and epimerases

#### Glutamate racemase (MurI)

Glutamate racemase catalyses the interconversion of *S*- and *R*-glutamate. *R*-Glutamate is a key component of the bacterial cell wall^17^ and the enzyme is an important drug target. Readers are also referred to the review on amino-acid racemases^20^ and the previous section for details of substrate/product analogues,^175^ allosteric inhibitors,^21,25,200^ covalent inhibitors,^22–24,134^ high-throughput screening^200^ and virtual screening.^23–25^

**Fig. 21:**
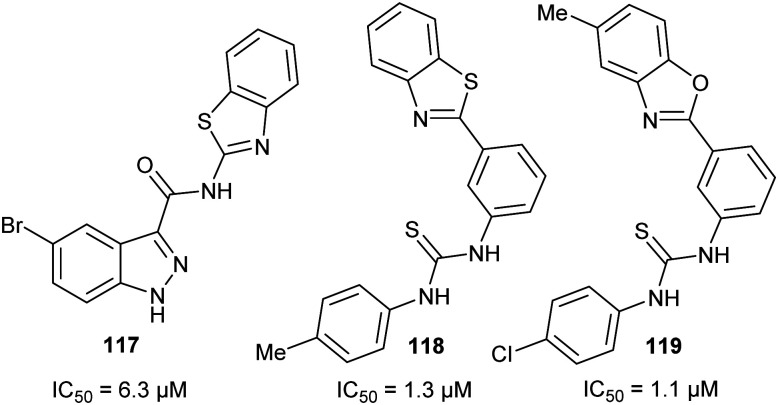
Structures of leads reported by Malapati *et al*.^246–248^

Malapati *et al.* have reported a series of medium-throughput screening studies on *M. tuberculosis* glutamate racemase using thermal-shift assays (Fig. 21).^246–248^ Structure–activity relationship (SAR) studies led to inhibitors **117–119** with low μM IC_50_ values. Non-competitive inhibition was assigned based on the observed changes within the thermal shift assay, although this was not confirmed by enzyme activity assays. Docking studies suggested that these compounds bound to an allosteric binding site.

**Fig. 22:**
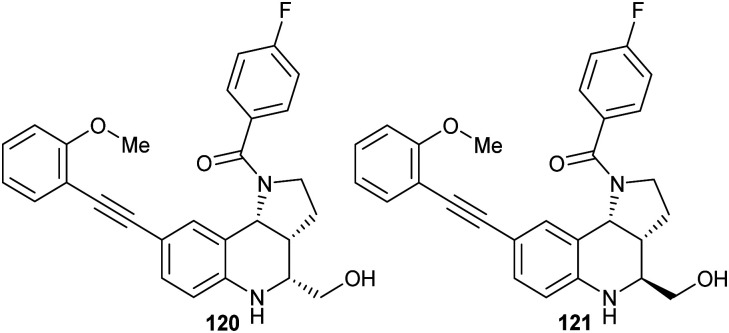
Structures of BRD0761 **120** and BRD3141 **121**.^249^

In addition, Duvall *et al.* reported phenotypic screening of a diversity-orientated synthetic collection (∼100 000 compounds) against *Clostridium difficile* and other bacterial strains under anaerobic conditions.^249^ One of the hits (BRD0761, Fig. 22, **120**) showed minimum inhibitory concentrations of 0.06–0.25 μg mL^−1^ (0.13–0.55 μM) against various *C. difficile* strains, with much higher MIC values against other anaerobes, while its epimer BRD3141 **121** was also active (Fig. 22). BRD0761 inhibited uptake of [^14^C]-*N*-acetylglucosamine into bacteria in a dose-dependent manner, suggesting that it targeted bacterial cell wall biosynthesis. The target was identified from resistance mutants as glutamate racemase and a binding model was produced based on the X-ray crystal structure of *H. pylori* enzyme. Dosing of mice with **120** protected them from *C. difficile* infection.

#### UDP-*N*-acetylglucosamine 2-epimerase

UDP-*N*-acetylglucosamine 2-epimerase is one of the first enzymes in the teichoic acid biosynthetic pathway,^250^ which is required for the integrity of the bacterial cell wall. In addition, Zika virus uses 2,3-linked sialic acid residues to enter mammalian cells and CRISPR-Cas9 knock-out of this enzyme reduces viral infection.^251^ The use of *N*-acetylmannosamine analogues as inhibitors is especially favourable, as *N*-acetylmannosamine is used solely for biosynthesis of sialic acid; in contrast, UDP-*N*-acetylglucosamine is also used in the biosynthesis of other glycans^252^ and analogues are likely to suffer from lack of selectivity.

**Fig. 23:**
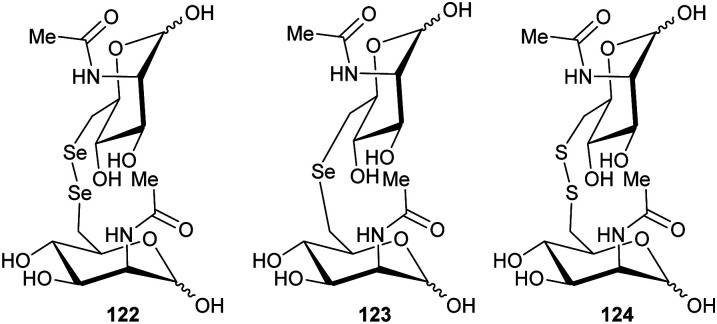
Structures of (di)selenide and disulfide inhibitors of UDP-*N*-acetylglucosamine 2-epimerase/*N*-acetylmannosamine kinase.^253^

A series of *N*-acetylglucosamines and *N*-acetylmannosamines, some with modified UDP moieties, have been previously developed as inhibitors but had modest potency (reviewed in ref. 253). Nieto-Garcia *et al.*, reported a series of inhibitors in which the C6 hydroxy group was replaced with sulfur or selenium (Fig. 23).^253^ The diselenide inhibitor **122** proved to be highly potent (IC_50_ = 8.5 μM) compared to the other inhibitors (IC_50_ values of 1.9 to >10 mM). The dimeric monoselenide inhibitor **123** was much less potent (IC_50_ = 3.0 mM). The corresponding disulfide analogue **124** was also much less active than **122** (IC_50_ = 4.2 mM), showing the importance of the diselenide unit for potent inhibition. The much higher potency of **122** compared to the other inhibitors could be due to bond length or flexibility of the linker.^253^ Small-molecule diselenide bonds have been reported as having a bond length of 2.29 Å,^254^ while disulfide bonds (in proteins) have a corresponding bond length of 2.05 Å.^255^ It has also been suggested that van der Waals interactions and hydrogen bonding potential may also be important in determining inhibitory potency.^253^ Diselenide **122** was a competitive inhibitor with a *K*_i_ value of 15.7 μM.^253^

**Fig. 24:**
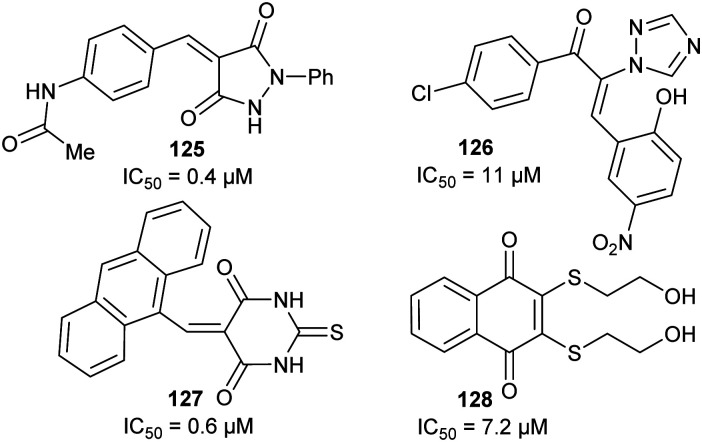
Structures of UDP-*N*-acetylglucosamine 2-epimerase/*N*-acetylmannosamine kinase inhibitors.^252^

Hinderlich *et al.* reported a high-throughput screening campaign using a library of 41 536 compounds and a luciferase assay to measure ATP depletion.^252^ The *N*-acetylmannosamine substrate was used at 33 μM, close to its *K*_m_ value, with an average *Z*′ value (a measure of the ability of the assay to discriminate between a hit and random noise^227,256^) of 0.78. Compounds were screened at 13 μM, yielding 252 hits of which 174 were analysed using dose–response curves, yielding 46 inhibitors with IC_50_ < 33 μM. Further analysis and counter-screening against yeast hexokinase yielded several leads **125–128** (Fig. 24). The IC_50_ values did not significantly change with changing concentrations of ATP, suggesting that they were non-competitive. Modelling studies suggested that **125**, **126** and **128** bound in the *N*-acetylmannosamine-binding site in the closed form of the enzyme, although **127** was larger than the available site, suggesting it might be bound to the open conformation.^252^

**Fig. 25:**
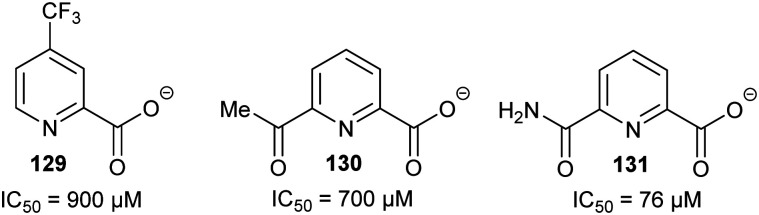
Structure of hit **129** and derived inhibitors **130** and **131**.^257^

UDP-*N*-acetylglucosamine epimerase/*N*-acetylmannosamine kinase is also one of only two racemases or epimerases to be subjected to a fragment-screening campaign.^257^ A library of 281 fluorinated fragments were screened at 50 μM using ^19^F NMR and binding of inhibitor was confirmed by competition with *N*-acetylmannosamine and ATP, yielding 23 hits. Of these, compound **129** was also shown to inhibit in a coupled enzyme assay and so was chosen for development. Analogues of **129** were screened, leading to identification of **130** which was optimised to **131** (Fig. 25). Modelling of the binding of the inhibitor suggested that **131** bound to the active-site Mg^2+^ used in the kinase reaction, near the catalytic site. However, these compounds were not developed into more potent leads.

#### dTDP-4-keto-6-deoxyglucose 3,5-epimerase (RmlC)

dTDP-4-keto-6-deoxyglucose 3,5-epimerase (RmlC) is involved in biosynthesis of l-rhamnose in *M. tuberculosis* and other bacteria.^111,228^l-Rhamnose is biosynthesised from d-glucose-6-phosphate in a four-step pathway. The third step of this pathway is epimerisation at both carbons C3 and C5 of the 4-ketosugar moiety catalysed by RmlC, followed by reduction of the keto group by RmlD in the final step.^111^ Because l-Rhamnose is essential for the integrity of the bacterial cell wall, RmlC and the other enzymes in the pathway are drug targets.^258–260^ RmlC is also responsible for activation of the virulence factor in the marine pathogen *Vibrio vulnificus*.^261^

**Fig. 26:**
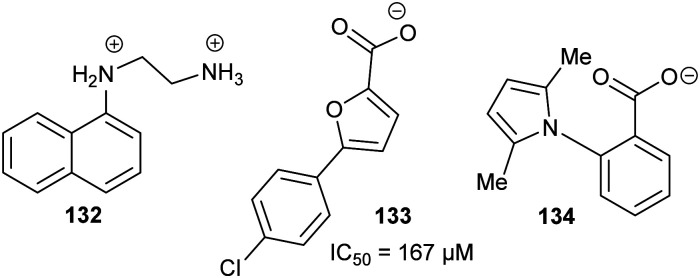
Structures of fragment screening hits active against RmlC.^259^

Several inhibitors of RmlC have been previously characterised (including the high-throughput screening inhibitors noted above;^228^*vide supra*, Fig. 16), although many have limited aqueous solubility.^259^ van der Beek *et al.* conducted a fragment-screening campaign with a commercial library using bio-layer interferometry.^259^ A library of ∼1000 fragments was screened at 200 μM with twelve hits. Of these, seven compounds showed dose-dependent enzyme inhibition and inhibited bacterial growth. Three hits (Fig. 26, **132–134**) with diverse structures inhibited both RmlB (the preceding enzyme in the biosynthetic pathway) and RmlC.

Sasikala *et al.* also conducted a virtual screen of RmlC and identified several potential inhibitors of the *Vibrio vulnificus* enzyme (also known as WbpP).^261^ However, none of these compounds were confirmed as hits in biochemical or biophysical screens.

#### α-Methylacyl-CoA racemase

α-Methylacyl-CoA racemase (AMACR; P504S) is a metabolic enzyme involved in the degradation of branched-chain fatty acids and the activation of ibuprofen and related drugs.^6,7^ Levels of the AMACR protein are increased in prostate cancer and many other cancers and the reader is referred to previous reviews on the subject.^6,7^ The M9V single-nucleotide polymorphism (SNP) is well known to increase risk of prostate cancer (reviewed in ref. 7) but recent analysis showed interaction of this SNP with SNPs in serine/threonine kinase AKT1 which are also involved in prostate cancer.^262^ Levels of AMACR protein have also been shown to be downregulated by microRNA miR200, resulting in decreased proliferation and migration of prostate cancer cells.^263^ Interestingly, a recent epidemiological study showed that AMACR levels were diminished in men with prostate cancer who supplemented their diet with extracts from cruciferous vegetables, such as broccoli, which contains the isothiocyanate compound, sulforaphane.^264^ AMACR levels are also increased in glioblastoma^265,266^ and high AMACR levels are correlated with poor prognosis for patients.^265^ Hence, AMACR is a potentially a novel biomarker for glioblastoma.^265^ siRNA knock-down of AMACR levels led to reduced proliferation of glioblastoma cells.^265^ Increased AMACR levels are thought to indicate an increase in fatty acid β-oxidation, in a similar way to that observed in prostate cancer.^266^

**Fig. 27:**
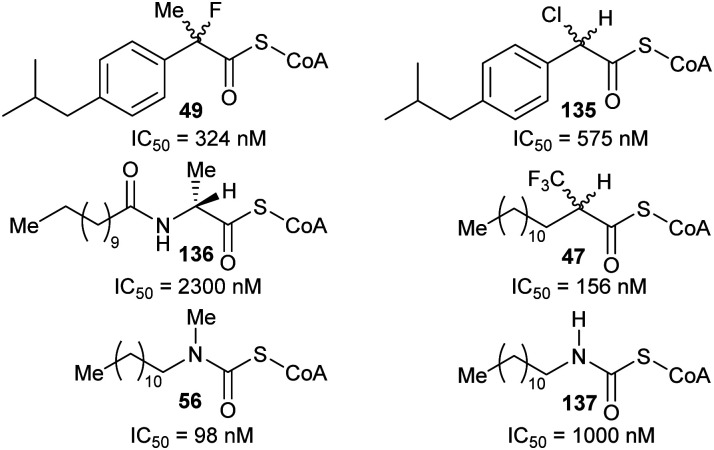
Structures of rationally designed AMACR inhibitors reported by Carnell *et al*.^191^

AMACR has been the subject of several previous inhibitor studies as well as structural studies on the *M. tuberculosis* homologue (MCR), with literature up to the end of 2012 having been previously reviewed.^6,7^ Following on from previous reports,^136^ Carnell *et al.* reported a series of acyl-CoA inhibitors with modified cores.^191^ The reported several new inhibitors (Fig. 27) including (±)-α-fluoroibuprofenoyl-CoA **49** (in which the C_α_–H was replaced by fluorine), a chloro derivative **135**, and *N*-dodecanoyl-*R*-alanyl-CoA **136**. Inhibitor **49** replaces the C_α_–H with a C_α_-F, effectively removing the α-proton. Substitution of hydrogen with fluorine is commonly used in drug design because of the similar atomic radii (1.10 *vs.* 1.35 Å),^267^ bond lengths to carbon (1.08 to 1.11 *vs.* 1.26 to 1.41 Å)^267^ and the high C-F bond energies (typically >456 kJ mol^−1^).^138,268^ Inhibitors **135** and **136** are expected to form the enolate intermediate more easily,^191^ although this was not actually proven. Model studies suggest the C_α_–H p*K*_a_ for **136** should be ∼14.5–16.5^86,191^ compared to a C_α_–H p*K*_a_ of ∼21 for standard acyl-CoA esters.^86,88^ Inhibitors **49** and **135** had IC_50_ values of 324 and 570 nM.^191^*N*-Dodecanoyl-*R*-alanyl-CoA **136** was less potent, with an IC_50_ value of 2300 nM, which is probably be due to lower stabilisation of the negatively charged intermediate.^191^ The known inhibitor (±)-2-trifluoromethyltetradecanoyl-CoA^136^**47** had an IC_50_ of 156 nM.^191^ Significantly, two potent carbamate inhibitors **56** and **137** as analogues of the intermediate enolate were reported (IC_50_ = 98 and 1000 nM). Later studies^127,164^ showed that the carbamate inhibitor **56** was highly potent compared to other inhibitors (IC_50_ = ∼0.4 nM using the colorimetric assay^127^).

**Fig. 28:**
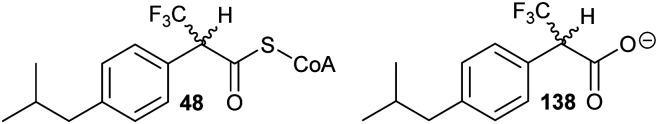
Structures of trifluoroibuprofenoyl-CoA **48** and the trifluoroibuprofen pro-drug **138**.^190^

Also following on from the Carnell *et al.* study in 2007,^136^ Festuccia *et al.*^190^ reported the synthesis and testing of trifluoroibuprofenoyl-CoA (Fig. 28, **48**). Limited kinetic analysis suggested non-competitive inhibition by this compound with a *K*_i_ = 1.7 μM. This result is notable because non-competitive inhibition of enzymes is rather rare (reviewed in ref. 227) and this is the only example of a non-competitive inhibitor reported for AMACR (and one of only a few for racemases/epimerases in general). Non-competitive inhibition is inconsistent with the inhibitor acting as an alternative substrate but instead arises through allosteric inhibition, stabilisation of an inactive conformation or covalent modification of the target.^227^ The basis for inhibition of AMACR by trifluoroibuprofenoyl-CoA is unclear, although elimination of fluoride is not reported. Treatment of cultured androgen-dependent and -independent prostate cancer cells with the pro-drug trifluoroibuprofen (Fig. 28, **138**) resulted in arrest at G2/M in the cell cycle and a host of other changes, including induction of apoptosis.^190^ Tumour growth in androgen-dependent and -independent prostate cancer xenograft mouse models was also significantly reduced by treatment with this agent.^190^

**Fig. 29:**
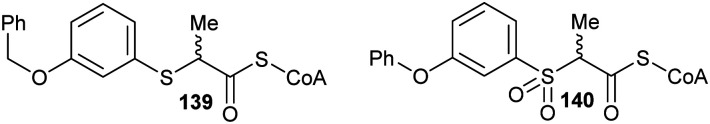
Structure of the most potent 2-(arylthio)propanoyl-CoA inhibitor **139** and the poorly active 2-(arylsulfonyl)propanoyl-CoA **140** of human AMACR.^165^

The advent of the AMACR colorimetric assay (*vide supra*, Scheme 9)^127^ has enabled much more thorough testing of inhibitors than had been previously possible, including determination of IC_50_ and *K*_i_ values and of reversibility of inhibition. This also enabled the first structure–activity relationship studies to be conducted. The first studies^127,164^ looked at a series of known AMACR inhibitors and substrates. A second study^165^ looked at a focussed series of 2-(arylthio)propanoyl-CoA inhibitors; the presence of the side-chain sulfur atom resulted in increased acidity of the C_α_–H (previous studies on straight-chain acyl-CoAs and their 3-thia analogues showed that the presence of the sulfur reduces the p*K*_a_ of the C_α_–H to ∼15–16.5,^269,270^ compared to ∼21 for the corresponding acyl-CoA^86,88^). Many of these 2-(arylthio)propanoyl-CoA inhibitors were equipotent to fenoprofenoyl-CoA but optimisation of the inhibitor side-chain resulted in increased potency, *e.g.***139**, IC_50_ = 22.3 nM.^165^ A 2-(arylsulfonyl)propanoyl-CoA inhibitor **140** was also synthesised in the hope that the presence of the sulfonyl group would further increase C_α_–H acidity but this proved to be a poor inhibitor (Fig. 29).^165^

**Fig. 30:**
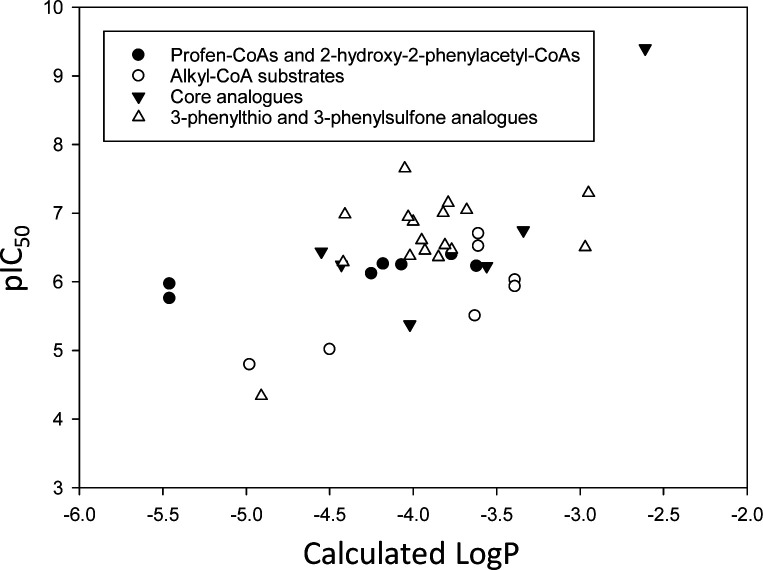
Potency of acyl-CoA inhibitors of AMACR, as measured by pIC_50_ as a function of calculated log *P* values. Inhibitors are as follows (with compound numbers from the original papers in parentheses): Ibuprofenoyl-CoA and analogues (5–11);^164^ straight-chain acyl-CoAs and other substrates (12–14 and 18–21);^164^ inhibitors with modified acyl-CoA cores (4, 22–26);^164^ and 2-arylthiapropanoyl-CoAs and 2-arylsulfonylpropanoyl-CoA (**7a–7n**, **10b**).^165^ Log *P* values were calculated using: https://www.molinspiration.com/cgi-bin/properties. Log *P*, log_10_ (ratio of concentrations of drug in octan-1-ol and water at equilibrium); pIC_50_, −log_10_IC_50_.

Plotting pIC_50_ values for all inhibitors^127,164,165^ characterised by the AMACR colorimetric assay^127^ against calculated log *P* values (Fig. 30) showed that inhibitor potency was positively correlated with log *P*. The 2-(arylsulfonyl)propanoyl-CoA inhibitor **140** was highly hydrophilic,^165^ suggesting that this was the reason for its unexpected low potency. Although the 2-(arylthio)propanoyl-CoA inhibitors, such as **139**, were highly potent in enzyme assays *in vitro* (IC_50_ = 22–520 nM), the carboxylic acid pro-drugs did not show any appreciable inhibition of androgen-dependent or -independent prostate cancer cells,^165^ possibly due to oxidation of the inhibitor pro-drug sulfur to the sulfoxide or sulfone.

**Fig. 31:**
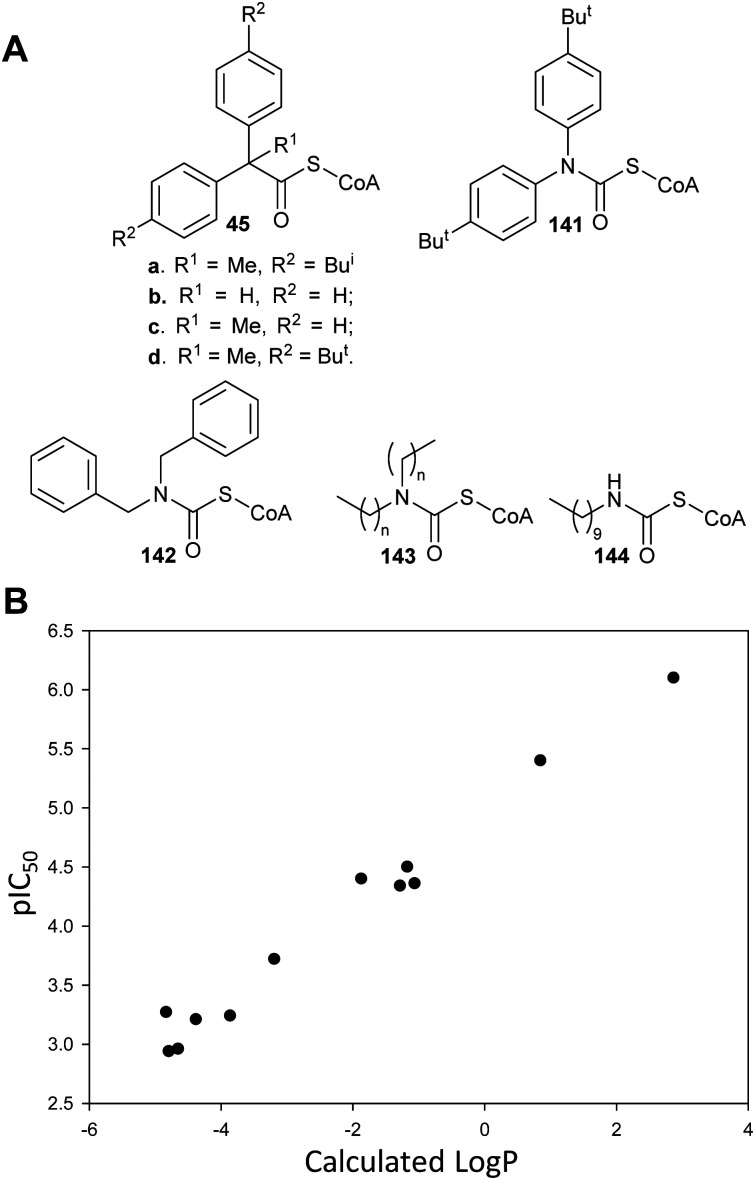
(A) Structures of substrate–product analogues^149,150^ inhibiting the *M. tuberculosis* homologue of AMACR (MCR). For **143**, *n* = 3, 5, 7, 9 or 11; (B) acyl-CoA inhibitor potency as measured by pIC_50_ as a function of calculated Log *P* values. Log *P* values were calculated using: https://www.molinspiration.com/cgi-bin/properties. Log *P*, log_10_ (ratio of concentrations of drug in octan-1-ol and water at equilibrium); pIC_50_, −log_10_IC_50_.

Since the last review,^7^ two studies featuring rational design of inhibitors for the *M. tuberculosis* AMACR homologue, MCR, have been published. The first study^149^ describes the synthesis and testing of several substrate/product acyl-CoA inhibitors (*vide supra*), in which the α-proton is replaced by a second side-chain in the inhibitor. The presence of the second sidechain increases potency of inhibition by ∼6-fold, although the measured absolute potency is relatively modest (*e.g.* 16.9 *cf.* 106 μM for **45a***vs.* ibuprofenoyl-CoA). One of these inhibitors (Fig. 31A, **45b**) has a α-proton in place of the α-methyl group and, as predicted, this does not undergo enzyme-catalysed exchange with solvent consistent with the α-proton being located in the methyl-binding site of the enzyme. The study is also notable in that several carboxylic acid precursors are also inhibitors, albeit with IC_50_ values in the mM range.^149^ Similar to the above AMACR inhibitors (*vide supra*, Fig. 30), potency of inhibition of MCR is also related to calculated log *P* values (Fig. 31).

Following from the observation that carbamate analogues are highly potent AMACR inhibitors,^127,164,191^ Pal *et al.*^150^ synthesised and tested carbamate analogues **141–144** of their substrate/product inhibitors against MCR (Fig. 31A).^149^ Inhibition is reported to be competitive, although the Lineweaver–Burk plots for some analogues, *e.g.***143** (*n* = 5), suggested mixed competitive inhibition. Surprisingly, several of these analogues show irreversible inhibition, in marked contrast to the carbamate AMACR inhibitors which are fully reversible.^127,164^ Inhibitors with long alkyl chains (*n* = 9 and 11) show saturating loss of activity with maximum *k*_inact_ values of ∼0.4 min^−1^ consistent with being active-site directed. Analogues with less lipophilic side-chains (**143**, *n* = 3) or a single side-chain (**144**) showed a non-saturating loss of enzymatic activity with a rate constant of 0.016–0.04 min^−1^.^150^ Inhibition was not reversed upon dialysis but no protein modification was observed by mass spectrometry. This observation is consistent with either non-covalent slow-binding inhibition, resulting in a long-lived enzyme-inhibitor complex, or irreversible inhibition resulting in a covalent modification of the protein, which is labile under mass spectrometric conditions.

Identification of AMACR inhibitors by high-throughput screening has also been reported.^163^ Unlike the previous study,^152^ the identified inhibitors were not non-specific protein modification agents.^163^ A number of pyrazoloquinolines and pyrazolopyrimidines were identified (*vide supra*, Fig. 15, **85a**, **85b** and **86**), and some structure–activity relationships were observed.^163^ The identified inhibitors displayed either mixed competitive or uncompetitive inhibition. The latter is a rare type of inhibition and arises from binding of inhibitor to the enzyme-substrate complex.

## Conclusions

Racemases and epimerases occupy a unique position in metabolism, in that they are the only major class of enzymes which can use substrates with both configurations at a chiral centre. Because of this, many racemases and epimerases are excellent drug targets and several have been extensively investigated as such, *e.g.* glutamate racemase.^20–25,134,175,200,249^ Use of inhibitors with the same configuration as the less abundant substrate (often d- or *R*-enantiomer) potentially offers additional benefits in that these isomers may be less prone to off-target binding and may have reduced drug metabolism, with consequent reductions in toxicities and longer durations of action.

However, efforts to develop drugs targeted against racemases and epimerases have been largely limited to rational design campaigns, with the few notable exceptions detailed above. Development of inhibitors which are alternative substrates has met with limited success, in part because several of the effective inhibitors are rapidly depleted *in vivo* whilst the effects of less effective inhibitors are readily overcome by the physiological substrate. Moreover, these inhibitors are necessarily chiral and there had been a move away from chiral drugs towards drugs with fewer sp^3^ carbons.^227^ This is despite a growing realisation that the attrition rate is higher for ‘flatter’ drugs^271,272^ and that licenced drugs have a higher average proportion of sp^3^ centres than molecules published in *The Journal of Medicinal Chemistry*.^273^ Consequently there has been a more recent move towards structures with a higher proportion of sp^3^ and chiral centres.^274^ Similarly, racemase/epimerase inhibitors in which the C_α_–H is replaced or where deprotonation is made more difficult tend not to be highly potent. The use of substrate–product analogues as inhibitors has also met with variable success. This strategy tends to work relatively well for racemases and epimerases in which large changes in substrate side-chain position occur during the reaction. Enzymes catalysing reactions resulting in limited changes in side-chain position and/or with small or sterically hindered active sites tend to be poorly inhibited by this type of compound.

There are relatively few developed inhibitors which are analogues of the transition state/deprotonated intermediate, perhaps because the early inhibitors developed against proline racemase were not highly effective and one of these inhibitors was an unstable imine.^135^ Some covalent inhibitors have been developed by rational design or identified by screening techniques.^20,22–24,26,27,46,124,134,217,218^ There has been renewed interest in the development of covalent inhibitors in recent years, prompted by a number of covalent drugs coming into clinical use.^201,205,207,208^ Covalent drugs acting on racemases and epimerases have all been directed against enzymes using active-site cysteine thiols^20,22–24,26,27,46,124,134,217,218^ (amino-acid racemases and epimerases). Covalently reacting drugs containing electrophiles reacting with other active-site bases^205,208^ have been under-explored. Both transition-state analogues^135,193,194^ and covalent inhibitors^201,204–206,208^ offer the potential for high potency and long duration of action and are potentially fertile ground for the future development of inhibitors.

Screening approaches^227^ have also been under-used to identify novel inhibitors. There are only five high-throughput screening campaigns in the literature^152,163,200,228,252^ and only two fragment-screening campaigns.^257,259^ Almost all racemases and epimerases catalyse reversible reactions and this places restriction on these assays but these can be overcome by using an elimination substrate or irreversible coupling enzyme (see section on enzyme assays for examples). The use of coupling enzymes also enables assays based on fluorescence or absorbance to be used, which are readily adaptable to high-throughput screening formats.^227^ Direct assaying of racemase or epimerase activity may also be possible using fluorescence anisotropy to monitor ligand binding.^227^

Fragment screening using assays of enzyme activity^227,275–279^ or biophysical techniques^275–280^ (particularly X-ray crystallography^274–277,279–281^) hold significant promise, although the different screening techniques have advantages and disadvantages^280^ and different tendencies towards false positive and negative results.^279^ There is also a balance to be struck between fragment complexity and affinity to maximise chances of success.^279^ Screening of fragment libraries for direct identification of inhibitors is particularly appealing for enzymes with small, enclosed active sites, *e.g.* proline racemase,^26,27^ as the amino-acid substrates are small fragments themselves (*M*_w_ = 89–204 Da). There have been a number of studies on the screening of small fragments (which will generally have low affinity^279^), including one using virtual screening initially to triage compounds which resulted in a 40% hit rate for a very small fragment library (fifteen compounds).^282^ Similarly, fragment-based screening holds promise for development of inhibitors of enzymes with larger active sites,^227,275–280^ although there are challenges associated with identification of different fragments which bind simultaneously and also in the elaboration of fragment hits into leads.^274,283^

It is important that inhibitors produced by rational design, identified by screening and other approaches are fully characterised to determine if covalent modification of the target is occurring. There are examples of rationally designed racemase inhibitors intended to be reversible which appear to exert their effects by covalent modification of the racemase target.^150^ Several inhibitors identified by screening approaches^239,252,259^ could also potentially inhibit their targets by covalent modification. Unselective modification of off-target proteins or other biological molecules could give rise to significant toxicities.^201–205,207–209,215^ Therefore, it is important to balance this potential draw-back with the advantages of covalent inhibition.

## Abbreviations used

AET: 5-(1-Aminoethyl)tetrazole
AMACR: Human α-methylacyl-CoA racemase (a.k.a. P504S) spliced variant 1A
DprE: Decaprenylphosphoryl-β-d-ribose epimerase
*Ec*L-DER: *E. coli* l-aspartate/l-glutamate racemase
IAM 12614: *L. aggregata cis*-3-hydroxy-*S*-proline racemase/dehydratase
MCR: α-Methylacyl-CoA racemase from *M. tuberculosis*
McyF: *Microcystis aeruginosa* aspartate racemase
MMP0739: Aspartate/glutamate racemase from *Methanococcus maripaludis*
Ph: phenyl
pIC_50_: −Log_10_(IC_50_)
PLP: Pyridoxal 5′-phosphate
RacX: *B. subtilis* arginine, lysine and ornithine racemase
RmlC: Deoxythymidine diphosphate-4-keto-6-deoxyglucose 3,5-epimerase
YgeA: *E. coli* homoserine racemase
YcjG: *E. coli* alanyl dipeptide epimerase. Standard one- and three-letter amino-acid codes are used.

## Conflicts of interest

There are no conflicts of interest to declare.
